# Hsp90 Governs Echinocandin Resistance in the Pathogenic Yeast *Candida albicans* via Calcineurin

**DOI:** 10.1371/journal.ppat.1000532

**Published:** 2009-07-31

**Authors:** Sheena D. Singh, Nicole Robbins, Aimee K. Zaas, Wiley A. Schell, John R. Perfect, Leah E. Cowen

**Affiliations:** 1 Department of Molecular Genetics, University of Toronto, Toronto, Ontario, Canada; 2 Department of Medicine, Duke University Medical Center, Durham, North Carolina, United States of America; 3 Department of Molecular Genetics and Microbiology, Duke University Medical Center, Durham, North Carolina, United States of America; Carnegie Mellon University, United States of America

## Abstract

*Candida albicans* is the leading fungal pathogen of humans, causing life-threatening disease in immunocompromised individuals. Treatment of candidiasis is hampered by the limited number of antifungal drugs whose efficacy is compromised by host toxicity, fungistatic activity, and the emergence of drug resistance. We previously established that the molecular chaperone Hsp90, which regulates the form and function of diverse client proteins, potentiates resistance to the azoles in *C. albicans* and in the model yeast *Saccharomyces cerevisiae*. Genetic studies in *S. cerevisiae* revealed that Hsp90's role in azole resistance is to enable crucial cellular responses to the membrane stress exerted by azoles via the client protein calcineurin. Here, we demonstrate that Hsp90 governs cellular circuitry required for resistance to the only new class of antifungals to reach the clinic in decades, the echinocandins, which inhibit biosynthesis of a critical component of the fungal cell wall. Pharmacological or genetic impairment of Hsp90 function reduced tolerance of *C. albicans* laboratory strains and resistance of clinical isolates to the echinocandins and created a fungicidal combination. Compromising calcineurin function phenocopied compromising Hsp90 function. We established that calcineurin is an Hsp90 client protein in *C. albicans*: reciprocal co-immunoprecipitation validated physical interaction; Hsp90 inhibition blocked calcineurin activation; and calcineurin levels were depleted upon genetic reduction of Hsp90. The downstream effector of calcineurin, Crz1, played a partial role in mediating calcineurin-dependent stress responses activated by echinocandins. Hsp90's role in echinocandin resistance has therapeutic potential given that genetic compromise of *C. albicans HSP90* expression enhanced the efficacy of an echinocandin in a murine model of disseminated candidiasis. Our results identify the first Hsp90 client protein in *C. albicans*, establish an entirely new role for Hsp90 in mediating resistance to echinocandins, and demonstrate that targeting Hsp90 provides a promising therapeutic strategy for the treatment of life-threatening fungal disease.

## Author Summary

Fungal pathogens pose a serious threat to people with compromised immune systems. Chief among the opportunistic fungal pathogens is *Candida albicans*. Treatment of *C. albicans* infections remains challenging because there are very few effective drugs and the pathogen has evolved many strategies to survive drug exposure. The echinocandins are the only new class of antifungal drug to reach the clinic in decades and they block biosynthesis of an essential component of the fungal cell wall. We discovered that the molecular chaperone Hsp90, which is required for its client proteins in the cell to fold and function, governs the ability of *C. albicans* to survive exposure to echinocandins. Compromising Hsp90 function renders the echinocandins more effective at killing *C. albicans* laboratory strains and clinical isolates. Hsp90 orchestrates the crucial responses to cell wall stress exerted by the echinocandins by enabling the function of its client protein calcineurin, which allows the fungus to survive otherwise lethal conditions. Our results suggest that compromising Hsp90 function provides a powerful and much-needed strategy to render existing antifungal drugs more effective in the treatment of life-threatening fungal infections.

## Introduction

*Candida* species have intimate yet perilous connections with their human hosts. They are commensals of the human microbiota of the gastrointestinal tract, mucous membranes, and skin. They also rank as the most common causative agents of invasive fungal infections and are responsible for a broad spectrum of disease [1],[2]. For the immunocompetent individual, *Candida* infections are most often superficial in nature including thrush and vaginitis. For the immunocompromised individual, these opportunists are far more menacing, as they can disseminate and cause life-threatening systemic disease. *Candida albicans* is the most frequently encountered *Candida* species in the clinic and is the fourth most common cause of hospital acquired infectious disease with mortality rates approaching 50% [2],[3]. The frequency of fungal infections continues to increase in pace with the growing immunocompromised patient population, including individuals undergoing chemotherapy, transplantation of solid organs or hematopoietic stem cells, as well as those infected with HIV [4],[5].

Treatment of invasive fungal infections remains notoriously challenging, due in large part to the limited availability of clinically useful antifungal drugs. Fungi are eukaryotes and share close evolutionary relationships with their human hosts [6],[7]. This makes the identification of drug targets in fungi that do not have homologs of similar function and susceptibility to inhibition in humans a daunting task. Most antifungal drugs in clinical use target the biosynthesis or function of ergosterol, the predominant sterol of fungal membranes, or the biosynthesis of (1,3)-β-D-glucan, a critical component of the fungal cell wall [8],[9]. The azoles are the largest class of antifungal drugs in clinical use and have been deployed for several decades. They inhibit lanosterol 14α-demethylase, blocking ergosterol biosynthesis and resulting in the accumulation of a toxic sterol intermediate that disrupts membrane integrity and results in cell membrane stress. The echinocandins are the only new class of antifungal drug to be approved for clinical use in decades and inhibit (1,3)-β-D-glucan synthase, disrupting cell wall integrity and resulting in cell wall stress.

The efficacy of antifungal drugs can be hampered by fungistatic rather than fungicidal activity, by host toxicity, and by the emergence of drug resistance. The azoles are generally fungistatic against *Candida* species and many immunocompromised patients are on long-term treatment due to persistent infections or on prophylaxis to prevent future infections. This creates favorable conditions for the evolution of drug resistance. In experimental populations and clinical isolates, resistance often emerges by multiple mechanisms [8]–[10]. Resistance mechanisms that minimize the impact of the drug include overexpression of multidrug transporters or alterations of the target enzyme. Other mechanisms function to minimize drug toxicity, such as loss of function of Erg3 in the ergosterol biosynthesis pathway, which blocks the production of a toxic sterol that would otherwise accumulate when the azoles inhibit their target. Mechanisms that mitigate drug toxicity are often dependent upon cellular stress responses that are crucial for tolerance of the membrane stress exerted by azoles [8],[9]. Far less is known about resistance to echinocandins, at least in part due to their more recent approval for clinical use. The most common mechanism of echinocandin resistance is mutation of the drug target [11]. The (1,3)-β-D-glucan synthase complex consists of a regulatory subunit, Rho1, and a catalytic subunit encoded by *FKS1*, *FKS2*, and *FKS3*. Resistance is most commonly associated with characteristic mutations in *FKS1* that reduce sensitivity of the enzyme to inhibition by echinocandins [11]–[13]. While the echinocandins are thought to be fungicidal against *C. albicans*, this organism has the capacity for robust growth at high drug concentrations, known as the paradoxical effect [14]. *C. albicans* may utilize multiple cellular stress response pathways to tolerate cell wall stress induced by echinocandins including upregulation of other components of the cell wall as well as responses mediated by the cell wall integrity signaling pathway [15],[16].

A key regulator of cellular stress responses crucial for resistance to the azoles is the molecular chaperone Hsp90. Hsp90 is an essential chaperone that regulates the form and function of many key signal transducers [17]–[19]. Pharmacological inhibition of Hsp90 blocks the emergence of azole resistance in *C. albicans* and abrogates resistance of laboratory mutants and clinical isolates that evolved resistance in a human host [20],[21]. Impairing Hsp90 function converts the fungistatic azoles into a fungicidal combination and enhances the therapeutic efficacy of azoles in two metazoan models of disseminated *C. albicans* infection [22]. Hsp90's role in the emergence and maintenance of azole resistance is conserved in the model yeast *Saccharomyces cerevisiae* [21]. The key mediator of Hsp90-dependent azole resistance is calcineurin, a protein phosphatase that regulates crucial responses to environmental stress, including the membrane stress exerted by exposure to azoles [20],[21]. In both *S. cerevisiae* and *C. albicans*, compromising calcineurin phenocopies compromising Hsp90, reducing azole resistance of diverse mutants. In *S. cerevisiae*, Hsp90 interacts physically with the catalytic subunit of calcineurin keeping it stable and poised for activation [23]. High-throughput genomic and proteomic studies have mapped Hsp90 physical interactors in *S. cerevisiae* [24], while to date not a single Hsp90 client protein has been identified in *C. albicans*.

Given Hsp90's role in azole resistance, we postulated that this chaperone might also govern crucial responses to the cell wall stress exerted by echinocandins in *C. albicans*. We recently discovered that Hsp90 is required for the basal tolerance of *Aspergillus* species to echinocandins, which are fungistatic against *Aspergillus* species, and that Hsp90 inhibitors enhance the efficacy of echinocandins in an invertebrate model of *Aspergillus fumigatus* infection [21],[22]. *A. fumigatus* is the principal causal agent of invasive aspergillosis with alarming mortality rates up to 90% that still remain at 40% with the best current treatment options [25],[26]. Compromising calcineurin tracks with compromising Hsp90, enhancing the activity of echinocandins [27],[28]. While initial studies did not detect a role for Hsp90 in echinocandin resistance in *C. albicans* [21], there are two lines of evidence implicating the Hsp90 client protein calcineurin in mediating responses to cell wall stress in this pathogen. First, stimulation of chitin synthesis rescues *C. albicans* from echinocandins and this stimulation is mediated via calcineurin in concert with the cell wall integrity signaling pathway and the high osmolarity glycerol signaling pathway [15],[16]. Second, inhibition of calcineurin can block the paradoxical growth of *C. albicans* observed at elevated echinocandin concentrations [29]. Whether calcineurin mediates basal tolerance to echinocandins is unclear given that in one study, deletion of calcineurin enhanced the killing activity of an echinocandin [30], while in another study there was no effect [31]. Thus, if Hsp90 regulates calcineurin function, then it is poised to mediate crucial cellular responses to the echinocandins.

Here, we investigated Hsp90's role in tolerance to echinocandins in *C. albicans*. We found that pharmacological or genetic compromise of Hsp90 function reduced tolerance of laboratory strains to the echinocandins and created a fungicidal combination. Inhibition of Hsp90 also reduced resistance acquired by mutation in *FKS1* in both laboratory-derived mutants and clinical isolates that acquired resistance in a human host. Compromising calcineurin function phenocopied compromising Hsp90 function. Consistent with calcineurin being the key mediator of Hsp90-dependent echinocandin tolerance, we established that calcineurin is an Hsp90 client protein in *C. albicans*. The downstream effector of calcineurin, Crz1, played a partial role in mediating calcineurin-dependent stress responses that are activated by echinocandins. Hsp90's key role in governing crucial responses to cell wall stress exerted by echinocandins was not conserved in *S. cerevisiae*, emphasizing the importance of performing molecular studies in the pathogen. In a murine model of disseminated candidiasis, genetic impairment of *HSP90* expression enhanced the therapeutic efficacy of an echinocandin. Our findings identify the first Hsp90 client protein in *C. albicans* and establish an entirely new role for Hsp90 in mediating echinocandin resistance. Further, our results demonstrate that targeting Hsp90 provides a promising therapeutic strategy for the treatment of life-threatening disease.

## Results

### Hsp90 plays a crucial role in echinocandin tolerance of *Candida albicans*

**Figure 1:**
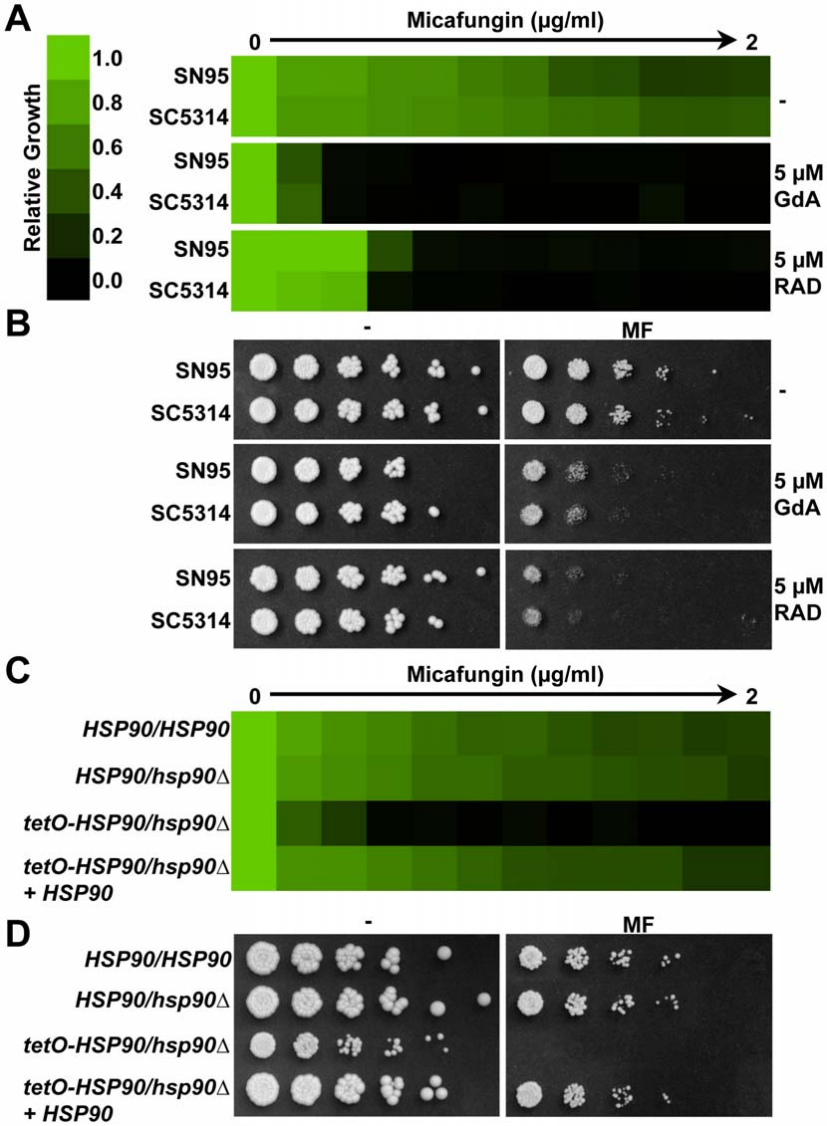
Hsp90 plays a crucial role in echinocandin tolerance of *Candida albicans*. (A) Pharmacological inhibition of Hsp90 with GdA or RAD reduces MF tolerance of *C. albicans* laboratory strains in an MIC assay. Assays were done in synthetic defined medium at 30°C for 72 hours. Optical densities were averaged for duplicate measurements and normalized relative to MF-free controls (see colour bar). (B) Pharmacological inhibition of Hsp90 reduces MF tolerance on solid rich medium (YPD). Cells were spotted in fivefold dilutions (from 1×10^6^ cells/ml) onto plates with a fixed concentration of MF (30 ng/ml), GdA, or RAD, as indicated, and were photographed after 48 hours in the dark at 30°C. (C) Genetic compromise of Hsp90 expression reduces MF tolerance in an MIC assay. The assay was performed and analyzed as in part A. (D) Genetic compromise of Hsp90 expression reduces MF tolerance on solid rich medium (YPD). The assay was performed and analyzed as in part B except plates were photographed after 72 hours.

To determine the impact of compromising Hsp90 function on tolerance to echinocandins, we first used two structurally unrelated inhibitors geldanamycin (GdA) or radicicol (RAD) that bind with high affinity to Hsp90's unusual adenosine triphosphate (ATP) binding pocket and inhibit ATP-dependent chaperone function [32],[33]. We used concentrations that abrogate resistance to azoles, but have no impact on growth on their own [20]–[22]. The impact of Hsp90 inhibitors on tolerance to the widely used echinocandin micafungin (MF) was evaluated using an antifungal susceptibility test that measures growth across a gradient of MF concentrations relative to a MF-free growth control. Both strains tested showed robust tolerance to MF (Figure 1A). Inhibition of Hsp90 with GdA or RAD dramatically enhanced sensitivity to MF in either synthetic defined medium (Figure 1A) or in rich medium (Figure S1A). Comparable effects were observed with another widely used echinocandin, caspofungin (CS, Figure S1B). The same trends were observed when a dilution series of cells was spotted on solid medium with a fixed concentration of MF; concentrations of Hsp90 inhibitors that had no impact on growth on their own enhanced susceptibility to MF (Figure 1B). Notably, while synergy of Hsp90 inhibitors with MF was observed in both liquid and solid media (Figure 1), the synergy with CS was restricted to liquid medium (data not shown). This explains why the synergy between Hsp90 inhibitors and echinocandins was not detected in a previous study, which used CS on solid medium [21]. The basis for the different responses with MF and CS on solid medium is unclear and the response with a third echinocandin, anidulafungin, remains to be determined. Strains were more sensitive to echinocandins in a medium used for clinical susceptibility testing (RPMI, Figure S1C and Figure S1D), however, compromising Hsp90 or calcineurin function further enhanced the sensitivity (Figure S1C).

Next, we exploited genetic regulation of Hsp90 to validate the impact of compromising Hsp90 function on echinocandin tolerance. Deletion of one *HSP90* allele had negligible effect on MF tolerance (Figure 1C and D). Replacing the native *HSP90* promoter of the heterozygote with a tetracycline-repressible promoter has no effect on basal Hsp90 levels in the absence of tetracycline at 30°C, but blocks induction of *HSP90* in response to stress such as elevated temperature of 37°C or exposure to antifungal drugs [22]. Even in the absence of tetracycline, compromising *HSP90* expression in the *tetO-HSP90/hsp90Δ* strain resulted in hypersensitivity to MF in both liquid and solid media (Figure 1C and 1D). While the *tetO-HSP90/hsp90Δ* strain also had a reduced growth rate, Hsp90 inhibitors at concentrations that have no effect on growth on their own dramatically enhanced echinocandin sensitivity ruling out the possibility that the hypersensitivity is simply due to reduced growth rate (Figure 1B). Restoring a wild-type *HSP90* allele to the *tetO-HSP90/hsp90Δ* strain complemented both the reduced growth rate and the hypersensitivity to MF. Thus, pharmacological and genetic studies establish that Hsp90 enables tolerance to echinocandins.

### Compromising calcineurin function phenocopies compromising Hsp90 function

It is now well established that a key mediator of Hsp90-dependent azole resistance is calcineurin, a protein phosphatase that regulates numerous responses to membrane stress in *C. albicans* [8],[20],[21]. If Hsp90 governs crucial cellular responses to echinocandins via calcineurin, then inhibition of calcineurin should phenocopy Hsp90 inhibition.

**Figure 2:**
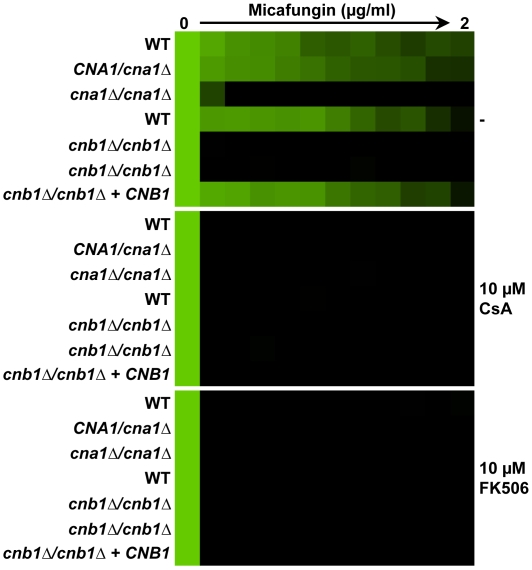
Compromising calcineurin function phenocopies compromising Hsp90 function. Deletion of either the catalytic subunit of calcineurin, *CNA1*, or the regulatory subunit of calcineurin, *CNB1*, abrogates MF tolerance. Pharmacological inhibition of calcineurin with either CsA or FK506 also abrogates MF tolerance. The assay was performed and analyzed as in Figure 1A.

We initially compromised calcineurin function pharmacologically using two structurally unrelated inhibitors cyclosporin A (CsA) and FK506 that inhibit calcineurin by distinct mechanisms [34]. CsA binds to Cpr1, a peptidyl-prolyl cis-trans isomerase (cyclophilin A), forming a drug-protein complex that blocks calcineurin function. FK506 forms a different drug-protein complex that binds to the structurally unrelated peptidyl-prolyl cis-trans isomerase FKBP12 to block calcineurin function. We used concentrations of CsA and FK506 that had no impact on growth on their own but that abrogate azole resistance [20],[21]. Inhibition of calcineurin with either CsA or FK506 abolished MF tolerance of *C. albicans* (Figure 2).

Next, we abolished calcineurin function genetically by either deleting the gene encoding the catalytic subunit of calcineurin, *CNA1*, or by deleting the gene encoding the regulatory subunit of calcineurin required for its activation, *CNB1*. In both cases, loss of calcineurin function abrogated MF tolerance (Figure 2). Reconstituting a wild-type allele of *CNB1* restored tolerance. Thus, impairing calcineurin function recapitulates the effects of impairing Hsp90, reducing echinocandin tolerance of *C. albicans*.

### Inhibition of Hsp90 or calcineurin creates a fungicidal combination with MF

The echinocandins are generally fungicidal against yeast species such as *C. albicans* [11]. However, *C. albicans* is able to grow vigorously at intermediate echinocandin concentrations in laboratory growth conditions (Figures 1 and 2 and Figure S1). Our previous assays did not resolve whether inhibition of Hsp90 or calcineurin results in a complete block in fungal growth in the presence of echinocandins or whether it creates a fungicidal condition.

**Figure 3:**
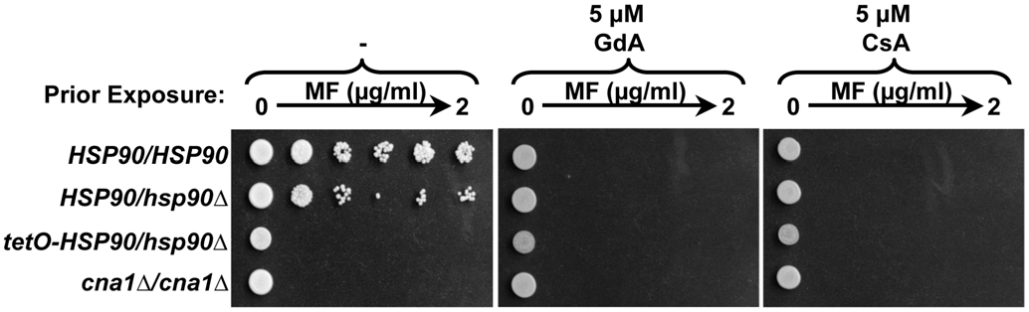
Inhibition of Hsp90 or calcineurin creates a fungicidal combination with MF. An MIC assay with four-fold MF dilutions was performed in YPD with or without either the Hsp90 inhibitor GdA or the calcineurin inhibitor CsA and incubated for 72 hours at 30°C. Cells from the MIC assay were spotted onto solid YPD medium and incubated at 30°C for 48 hours.

To determine if compromising Hsp90 or calcineurin function is fungistatic or fungicidal in the presence of echinocandins, we used tandem assays with an antifungal susceptibility test followed by spotting onto rich medium without any inhibitors. The common approach to address cidality by measuring colony forming units (CFU) in a culture exposed to treatment over time worked well for azoles [22], but was not accurate for echinocandins. Exposure of *C. albicans* to MF caused severe clumping such that large aggregates of cells were not separable, rendering CFU counts inaccurate (data not shown). A strain with wild-type or heterozygous *HSP90* levels was able to grow on rich medium following exposure to all concentrations of MF tested (Figure 3, left panel). Genetic compromise of *HSP90* expression in the *tetO-HSP90/hsp90Δ* strain or pharmacological inhibition of Hsp90 with GdA was cidal in combination with any dose of MF tested; no cells were able to grow on the rich medium following exposure to the treatments (Figure 3). Comparable effects were seen with genetic or pharmacological compromise of calcineurin function (Figure 3). Thus, Hsp90 and calcineurin regulate crucial cellular responses for surviving the cell wall stress exerted by the echinocandins.

### Calcineurin is an Hsp90 client protein in *C. albicans*

Compromising calcineurin pharmacologically or genetically phenocopies compromising Hsp90 suggesting a functional relationship between these regulators. Genetic studies established that calcineurin is a key mediator of Hsp90-dependent azole resistance [20],[21]. In *S. cerevisiae*, Hsp90 physically interacts with the catalytic subunit of calcineurin keeping it stable and poised for activation [23]. High-throughput studies have mapped Hsp90 physical interactors in *S. cerevisiae* [24], while to date not a single Hsp90 client protein has been characterized in *C. albicans*.

**Figure 4:**
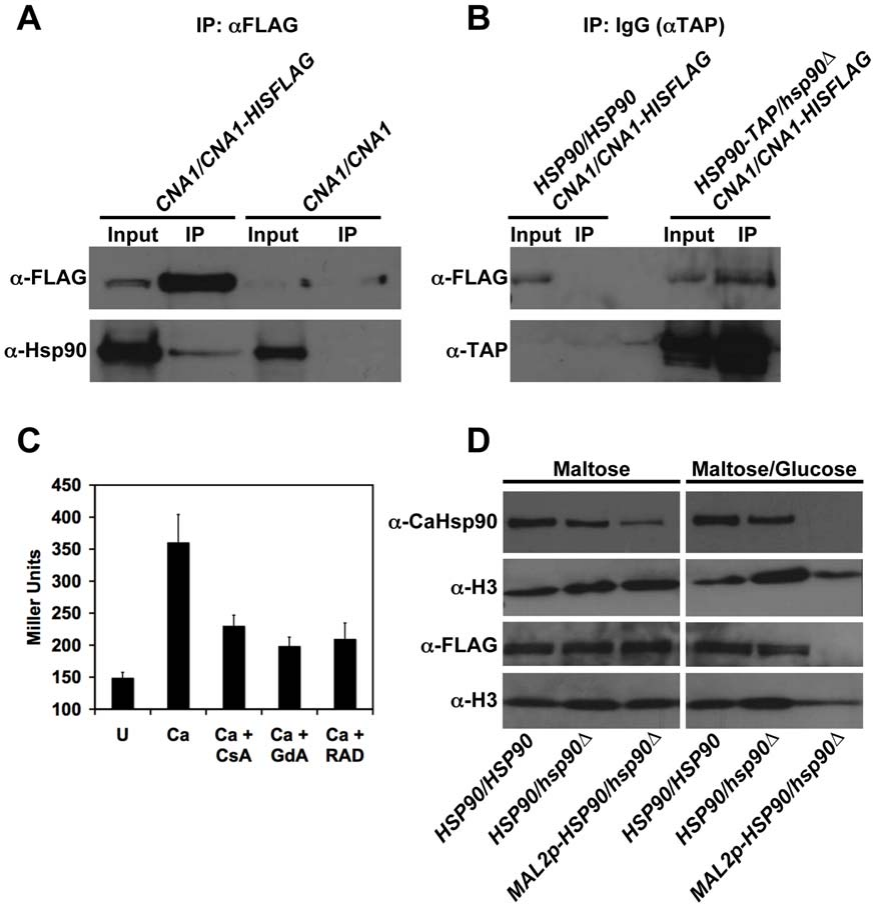
Calcineurin is an Hsp90 client protein in *C. albicans*. (A) Hsp90 and calcineurin physically interact as measured by co-immunoprecipitation of Hsp90 with Cna1-HisFLAG. Immunoprecipitation of HisFLAG-tagged Cna1 with anti-FLAG M2 affinity agarose, co-purifies Hsp90. Hsp90 was not immunoprecipitated by anti-FLAG M2 affinity agarose in control cells harboring untagged Cna1. (B) Hsp90 and calcineurin physically interact as measured by the reciprocal co-immunoprecipitation of Cna1-HisFLAG with Hsp90-TAP. Immunoprecipitation of Hsp90-TAP with IgG agarose, co-purifies Cna1-HisFLAG. Cna1-HisFLAG was not immunoprecipitated by IgG agarose in control cells harboring untagged Hsp90. (C) Calcineurin activation is blocked by pharmacological inhibition of Hsp90. A strain harboring a *UTR2p-lacZ* construct was incubated in rich medium with no treatment (U) or with 0.2 M CaCl_2_ (Ca) to activate calcineurin. The impact of the calcineurin inhibitor CsA (10 µM), or the Hsp90 inhibitors GdA (5 µM) or RAD (5 µM) on calcineurin activation was determined by measurement of β-galactosidase activity. Data are means±standard deviations for triplicate samples. (D) Genetic reduction of Hsp90 levels results in depletion of calcineurin. All strains shown in this panel have one allele of Cna1-HisFLAG in addition to the indicated genotype. Even when fully induced in the maltose, expression of Hsp90 from the *MAL2* promoter is not as strong as from the native promoter, while glucose results in further reduction of Hsp90 expression. This reduction of Hsp90 levels is accompanied by depletion of calcineurin. Top two panels, immune blot analysis of Hsp90 levels relative to the histone H3 loading control (5 µg protein loaded per well). Bottom two panels, immune blot analysis of Cna1-HisFLAG relative to the histone H3 loading control (50 µg protein loaded per well).

In order to determine if Hsp90 and calcineurin physically interact in *C. albicans*, we engineered strains harboring epitope-tagged proteins for co-immunoprecipitation. We tagged the catalytic subunit of calcineurin, Cna1, at the C-terminus using a 6X-histidine and FLAG epitope tag that has been used successfully for purification of the *C. albicans* septin complex [35]. The Cna1-His-FLAG protein is functional and sufficient to mediate the canonical calcineurin-dependent response to calcium stress (Figure S2A). Immunoprecipitation with anti-FLAG agarose co-purified both FLAG-tagged Cna1 and wild-type Hsp90 (Figure 4A). For the control strain lacking the tagged *CNA1* allele, Hsp90 was present in the input but was not immunoprecipitated. To further validate the physical interaction between Hsp90 and calcineurin, we performed the reciprocal co-immunoprecipitation using the same tagged allele of calcineurin in addition to an *HSP90* allele tagged at the C-terminus with a tandem affinity purification (TAP) tag which consists of a calmodulin binding peptide, a TEV cleavage site and two IgG binding domains of *Staphylococcus aureus* protein A that has been used with great success in *S. cerevisiae* [36]. The Hsp90-TAP protein is functional and able to support growth and all essential Hsp90 functions (Figure S2B). Immunoprecipitation with IgG sepharose for the TAP tag, co-purifies both Hsp90-TAP and Cna1-His-FLAG (Figure 4B). For the control strain lacking Hsp90-TAP, the tagged allele of calcineurin was present in the input but was not immunoprecipitated. Thus, reciprocal co-immunoprecipitation demonstrates physical interaction between Hsp90 and calcineurin in *C. albicans*.

If calcineurin is an Hsp90 client protein, then one would expect that inhibition of Hsp90 function would compromise calcineurin activation. To determine if this is indeed the case, we used a well-established reporter system that exploits the calcineurin downstream effector Crz1. Crz1 is a transcription factor that is dephosphorylated by calcineurin in response to calcineurin activation by calcium [37]–[39]. Dephosphorylated Crz1 translocates to the nucleus and drives expression of genes containing calcineurin-dependent response elements (CDREs) in their promoters. We used a strain harboring a construct with the *UTR2* promoter, which contains a CDRE element and is regulated by calcineurin [37], fused to *lacZ* and integrated at the *UTR2* locus [40]. In *S. cerevisiae*, a similar reporter that contains four tandem copies of CDRE and a *CYC1* minimal promoter driving *lacZ* has been used extensively [39]. As expected, exposure of cells containing the *UTR2-lacZ* reporter to calcium chloride resulted in activation of calcineurin relative to the untreated control (*P*<0.001, ANOVA, Bonferroni's Multiple Comparison Test Figure 4C). Inhibition of calcineurin with CsA caused a dramatic reduction of calcineurin activation (*P*<0.001). Inhibition of Hsp90 with GdA or RAD was as effective in blocking calcineurin activation as CsA (Figure 4C).

A hallmark of Hsp90 client proteins is that they are destabilized and degraded upon compromising Hsp90 function. To determine if calcineurin levels are reduced upon genetic reduction of Hsp90, we turned to a strain with its only *HSP90* allele regulated by the *MAL2* repressible promoter. In this system, *HSP90* expression is induced by maltose and repressed by glucose (Figure 4D). The *MAL2* promoter does not drive as strong expression as the native *HSP90* promoter, thus even when fully induced in maltose, the *MAL2p-HSP90/hsp90Δ* strain had a modest reduction of Hsp90 levels relative to a heterozygote with its only *HSP90* allele under the control of the native promoter (Figure 4D). Growth of cells in an equal mixture of glucose and maltose as the carbon source resulted in a dramatic reduction of Hsp90 levels (Figure 4D). Under these conditions, the *MAL2p-HSP90/hsp90Δ* strain has reduced growth rate and reaches approximately half the stationary phase cell density as a wild-type strain [40]. This genetic depletion of Hsp90 was accompanied by a dramatic reduction of calcineurin levels as measured by immunoblot hybridization with an anti-FLAG antibody to detect the Cna1-His-FLAG protein (Figure 4D). Hybridization with an anti-H3 antibody confirmed comparable amounts of protein were loaded for all strains. Taken together, these results support the model that calcineurin is a client protein in *C. albicans*.

### Azoles and echinocandins activate calcineurin-dependent stress responses

**Figure 5:**
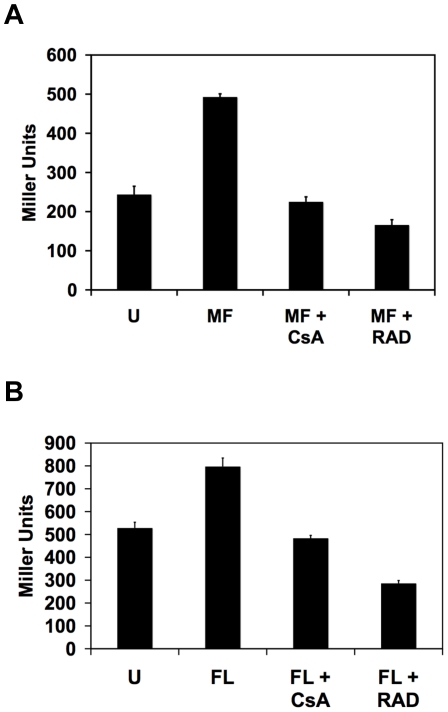
Echinocandins and azoles activate calcineurin-dependent stress responses. (A) The echinocandin MF activates calcineurin. A strain harboring a *UTR2p-lacZ* construct was incubated in rich medium without treatment (U) or with 30 ng/ml MF in combination with the calcineurin inhibitor CsA (10 µM) or the Hsp90 inhibitor RAD (5 µM), as indicated, for 8 hours. Data are means±standard deviations for triplicate samples. (B) The azole fluconazole (FL) activates calcineurin in an Hsp90-dependent manner. A strain harboring a *UTR2p-lacZ* construct was incubated in rich medium without treatment (U) or with 16 µg/ml FL in combination with the calcineurin inhibitor CsA (10 µM) or the Hsp90 inhibitor RAD (5 µM), as indicated, for 24 hours.

Due to the important role of calcineurin in mediating crucial responses to the stress exerted by exposure to azoles and echinocandins [20],[21],[30],[31], we postulated that these drugs would cause activation of calcineurin. We used the *UTR2p-lacZ* reporter to monitor calcineurin activation in response to concentrations of the azole antifungal drug fluconazole (FL) and the echinocandin MF that each cause modest inhibition of growth. Preliminary studies revealed maximum activation of calcineurin occurred at different time points in response to the different drugs (data not shown). Exposure to MF for 8 hours caused significant activation of calcineurin (Figure 5A, *P*<0.001, ANOVA, Bonferroni's Multiple Comparison Test). Pharmacological inhibition of calcineurin or Hsp90 blocked MF-induced calcineurin activation (*P*<0.001). Treatment conditions were optimized such that all cultures underwent comparable growth with equivalent protein yields. Exposure to FL for 24 hours also led to significant calcineurin activation (Figure 5B, *P*<0.001, ANOVA, Bonferroni's Multiple Comparison Test). Inhibition of calcineurin or Hsp90 blocked FL-induced calcineurin activation (Figure 5B, *P*<0.001). Thus, both echinocandins and azoles activate calcineurin-dependent stress responses mediated via the transcription factor Crz1 and inhibition of Hsp90 blocks these responses.

### The calcineurin-dependent transcription factor Crz1 plays a partial role in echinocandin tolerance

**Figure 6:**
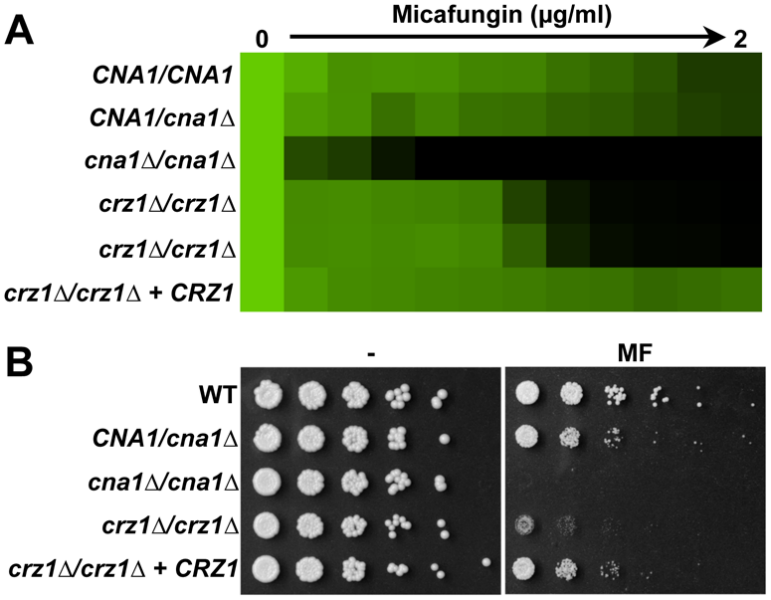
The calcineurin-dependent transcription factor Crz1 plays a partial role in echinocandin tolerance. (A) Homozygous deletion of *CRZ1* partially reduces tolerance to MF in an MIC assay. The assay was performed and analyzed as in Figure 1A. (B) Homozygous deletion of *CRZ1* partially reduces tolerance to MF on solid rich medium (YPD). Complementation with a wild-type *CRZ1* allele restores tolerance. The assay was performed and analyzed as in Figure 1B.

Crz1 is the key mediator of calcineurin-dependent transcriptional responses [37],[41] and is implicated in tolerance to azoles in both *S. cerevisiae* and *C. albicans* [20],[42]. While deletion of calcineurin causes a complete loss of azole tolerance, deletion of *CRZ1* causes only a partial reduction in both species. To determine if Crz1 is also an important effector of calcineurin-dependent echinocandin tolerance, we compared the phenotypic consequences of deletion of *CRZ1* with deletion of the catalytic subunit of calcineurin, *CNA1*. Mutants with homozygous deletion of *CNA1* were hypersensitive to MF in both liquid and solid assays (Figures 2 and 6). Two independent *crz1* null mutants demonstrated partial loss of MF tolerance, but were not as sensitive as the *cna1* mutants (Figure 6). Reconstitution of a wild-type *CRZ1* allele restored MF tolerance. Thus, Crz1 is a key mediator of calcineurin-dependent echinocandin tolerance, but other calcineurin downstream effectors affecting this trait remain to be identified.

### Clinical relevance of Hsp90 and calcineurin-mediated echinocandin resistance

**Figure 7:**
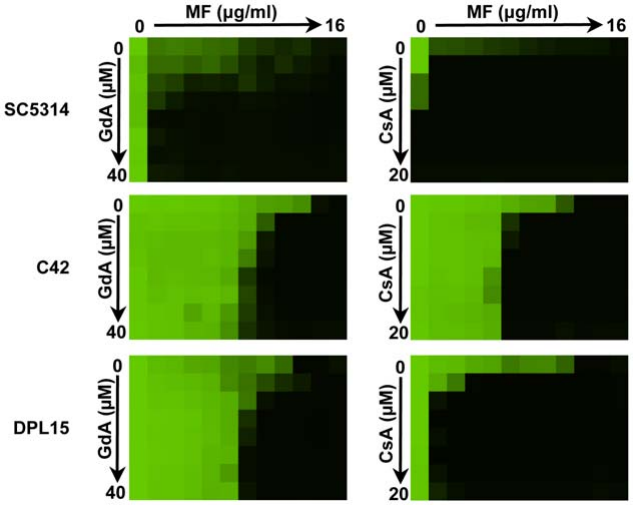
Hsp90 and calcineurin mediate echinocandin resistance of isolates that acquired Fks1 mutations during selection *in vitro* or in a human host. Pharmacological inhibition of Hsp90 or calcineurin reduces MF tolerance of a laboratory strain (SC5314), a laboratory derived Fks1 F641S mutant (C42), and a clinical isolate harboring the same Fks1 mutation (DPL15). Checkerboards were performed in synthetic defined medium and incubated at 30°C for 72 hours. Data was analyzed as in Figure 1A.

To determine if Hsp90 and calcineurin are involved in *bona fide* echinocandin resistance arising due to mutations in the target Fks1 we tested for synergy between inhibitors of Hsp90 (GdA) or calcineurin (CsA) and the echinocandin MF. We utilized a checkerboard format to explore a range of concentrations of each inhibitor to more accurately define the thresholds of synergy. For a standard laboratory strain, SC5314, potent synergy was observed such that very low concentrations of either GdA or CsA were sufficient to abrogate MF tolerance (Figure 7). Next, we tested an echinocandin resistant mutant that was selected *in vitro* in the SC5314 background by plating on a high concentration of the echinocandin caspofungin (CS) and contained the common Fks1 mutation F641S [12]. For this laboratory derived Fks1 mutant, C42, synergy was observed; GdA or CsA reduced MF resistance, though not to the same extent as for SC5314 (Figure 7). To determine if the synergy between GdA or CsA and MF was conserved in an isolate that evolved echinocandin resistance in a human host, we tested a clinical isolate harboring the same F641S Fks1 mutation (DPL15, generously provided by D. S. Perlin). Comparable synergy between GdA and MF was observed for both the clinical and laboratory-derived Fks1 mutants, however, the synergy between CsA and MF was more potent against the clinical isolate (Figure 7). Interestingly, these synergies were not observed for all echinocandin resistant clinical isolates tested, even those harboring the identical *FKS1* mutation; of the 14 *FKS1* mutants tested, synergy was observed for 8 (data not shown). These results suggest that Hsp90 and calcineurin enable cellular stress responses required for clinically relevant echinocandin resistance.

### Genetic compromise of *HSP90* expression enhanced the therapeutic efficacy of micafungin in a murine model of disseminated *C. albicans* infection

**Figure 8:**
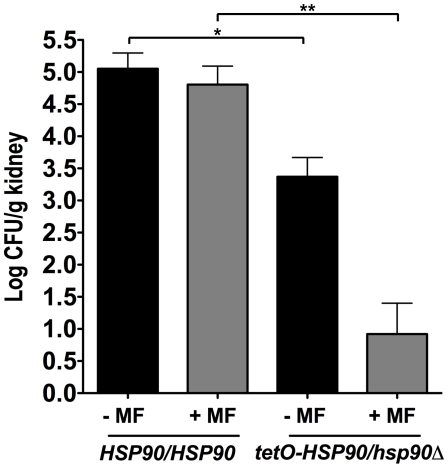
Genetic compromise of *C. albicans HSP90* renders micafungin (MF) more efficacious in a murine model of disseminated disease. CD1 mice were infected with an inoculum of 100 µl of 2×10^6^ colony forming units (CFU)/ml of a strain expressing wild-type *HSP90* levels or a strain with its only *HSP90* allele regulated by *tetO*. MF was administered at 0.2 mg/kg intraperitoneally at one-hour post infection and then daily, as indicated. One asterisk indicates *P*<0.05; two asterisks indicate *P*<0.001 (ANOVA, Bonferroni's Multiple Comparison Test).

To determine if impairing Hsp90 function holds therapeutic potential in combination with an echinocandin, we turned to a well-established murine model in which fungal inoculum is delivered by tail vein injection and progresses from the bloodstream to deep-seated infection of major organs such as the kidney [22],[40]. Due to toxicity of currently available Hsp90 inhibitors that do not distinguish pathogen from host in the context of an acute fungal infection [22], we used genetic regulation of *HSP90* to test this hypothesis in an *in vivo* system. We compared kidney fungal burden of mice infected with either a strain with wild-type *HSP90* levels or a strain with its only *HSP90* allele expressed under the *tetO* promoter. In the absence of tetracycline, the *tetO-HSP90/hsp90Δ* strain has *HSP90* levels comparable to a heterozygote but *HSP90* expression from the *tetO* promoter cannot be upregulated in response to host temperatures or drug stress [22]. Mice infected with the *tetO-HSP90/hsp90Δ* strain demonstrated significantly reduced kidney fungal burden relative to those infected with a strain expressing wild-type *HSP90* levels (*P*<0.05, ANOVA, Bonferroni's Multiple Comparison Test, Figure 8). Treatment of mice with a dose of MF that had negligible effect on mice infected with the strain with wild-type *HSP90* levels resulted in a significant reduction in fungal burden for mice infected with the *tetO-HSP90/hsp90Δ* strain (*P*<0.001, ANOVA, Bonferroni's Multiple Comparison Test, Figure 8). Thus, genetic compromise of *HSP90* expression enhances the efficacy of MF in a murine model.

### Divergence of Hsp90 and calcineurin's role in echinocandin tolerance in *S. cerevisiae*

**Figure 9:**
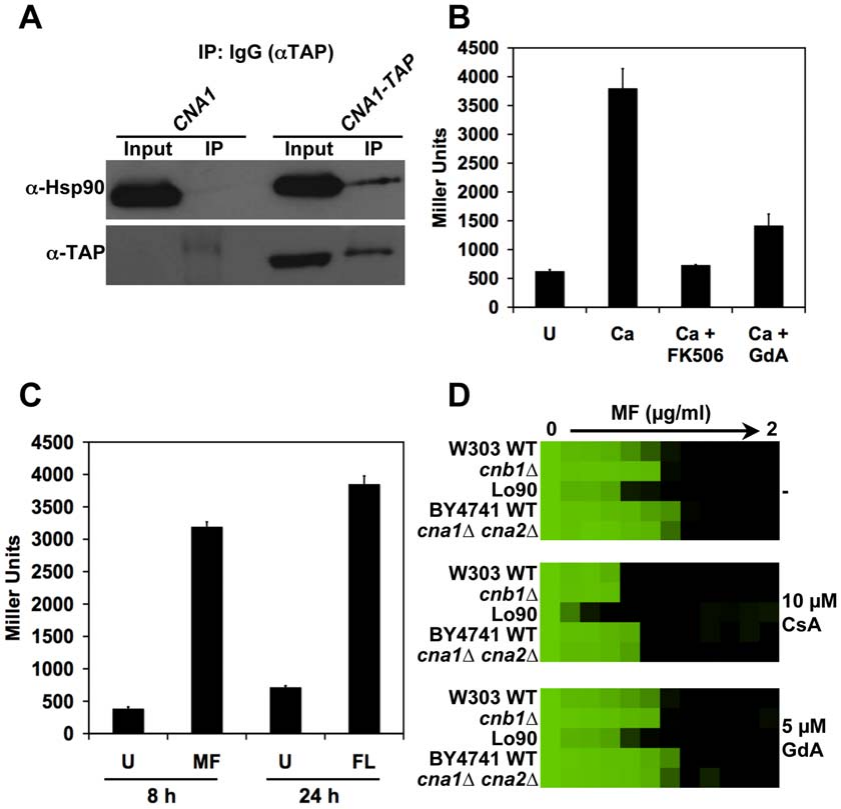
Divergence of Hsp90 and calcineurin's role in echinocandin tolerance in *S. cerevisiae*. (A) Hsp90 and calcineurin physically interact in *S. cerevisiae* as measured by co-immunoprecipitation of Hsp90 (encoded by *HSC82* and *HSP82*) with Cna1-TAP. Immunoprecipitation of TAP-tagged Cna1 with IgG agarose co-purifies Hsp90. Hsp90 was not immunoprecipitated by IgG agarose in control cells harboring untagged Cna1. (B) Calcineurin activation is blocked by pharmacological inhibition of Hsp90. A strain harboring a *CDRE-lacZ* construct was incubated in synthetic defined medium with no treatment (U) or with 0.2 M CaCl_2_ (Ca) to activate calcineurin. The impact of the calcineurin inhibitor FK506 (1 µg/ml) or the Hsp90 inhibitor GdA (5 µM) on calcineurin activation was determined by measurement of β-galactosidase activity. Data are means±standard deviations for triplicate samples. (C) The echinocandin MF and the azole FL activate calcineurin. A strain harboring a *CDRE-lacZ* construct was incubated in synthetic defined medium without treatment (U), with 30 ng/ml MF for 8 hours, or with 16 µg/ml FL for 24 hours. Data are means±standard deviations for triplicate samples. (D) Compromising calcineurin or Hsp90 has minimal effect on tolerance to MF in *S. cerevisiae* in an MIC assay. The effects of CsA are not due to inhibition of calcineurin given that genetic compromise of calcineurin by deletion of the regulatory subunit encoded by *CNB1* or by deletion of the catalytic subunit encoded by *CNA1* and *CNA2* has no impact on MF tolerance. The assay was performed in synthetic defined medium at 25°C and was analyzed as in Figure 1A.

Given that calcineurin is the key mediator of Hsp90-dependent resistance to azoles in both *S. cerevisiae* and *C. albicans*, we postulated that these key regulators of cellular signaling might also mediate tolerance to echinocandins in both species. Consistent with previous findings [23], we confirmed that Hsp90 and calcineurin physically interact in *S. cerevisiae* (Figure 9A), as they do in *C. albicans* (Figure 4). To monitor calcineurin activation in *S. cerevisiae*, we used a reporter system similar to that used for *C. albicans*. Cells contained an integrated plasmid with four tandem copies of CDRE and a *CYC1* minimal promoter driving *lacZ* [39]. As expected for an Hsp90 client protein, calcineurin activation was blocked upon pharmacological inhibition of Hsp90 (Figure 9B, *P*<0.001, ANOVA, Bonferroni's Multiple Comparison Test). FL activated calcineurin in *S. cerevisiae* (Figure 9C, *P*<0.0001, *t*-test), as it did with *C. albicans* (Figure 5B), consistent with the key role for both regulators in azole tolerance. MF also activated calcineurin in *S. cerevisiae* (Figure 9C, *P*<0.0001) as it did in *C. albicans* (Figure 5A).

Despite activation of calcineurin by MF in *S. cerevisiae*, compromise of calcineurin or Hsp90 function had negligible effect on MF tolerance. Neither deletion of the gene encoding the regulatory subunit Cnb1 nor deletion of the redundant genes encoding the catalytic subunit Cna1 and Cna2 reduced MF tolerance (Figure 9D). Consistent with this result, pharmacological inhibition of calcineurin with CsA had no impact on MF tolerance. A strain with genetically reduced Hsp90 levels (Lo90 [21]) had a modest reduction in tolerance, however, a concentration the Hsp90 inhibitor GdA that abrogates azole resistance had no effect on MF tolerance (Figure 9D). This suggests that the slight reduction in MF tolerance of the Lo90 strain may be due to a reduced growth rate rather than compromise of Hsp90 function. Thus, while the functional relationship between Hsp90 and calcineurin is conserved between *C. albicans* and *S. cerevisiae*, as is the activation of calcineurin in response to drug stress, these regulators play a crucial role in cellular responses to echinocandins in the pathogenic yeast but not in the model yeast.

## Discussion

Our results establish a new role for Hsp90 in echinocandin resistance in the pathogenic yeast *C. albicans*. Hsp90 regulates crucial cellular responses to the cell wall stress exerted by echinocandins such that compromising Hsp90 function reduces echinocandin tolerance of laboratory strains and resistance of clinical isolates (Figures 1 and 7). In a murine model of disseminated *C. albicans* infection, genetic compromise of *HSP90* enhances the efficacy of an echinocandin (Figure 8). We demonstrate that calcineurin is an Hsp90 client protein (Figure 4): calcineurin physically interacts with Hsp90; calcineurin activation is blocked upon impairment of Hsp90 function; and calcineurin levels are depleted upon genetic reduction of Hsp90. Our findings implicate calcineurin as the key mediator of Hsp90-dependent echinocandin resistance. Exposure to azoles and echinocandins activates calcineurin-dependent stress responses (Figure 5) and the downstream effector Crz1 plays a partial role in echinocandin tolerance (Figure 6). In addition to defining a novel mechanism of resistance to the only new class of antifungal drugs to reach the clinic in decades, these results provide the first characterization of an Hsp90 client protein in *C. albicans*.

The requirement for Hsp90 and calcineurin in mediating crucial cellular responses to the echinocandins in *C. albicans* but not in *S. cerevisiae* (Figures 1, 2, and 9) stands in contrast to the conserved role for both regulators in cellular responses to azoles in both species. It is intriguing that calcineurin is activated in response to echinocandin stress in *S. cerevisiae* yet the functional consequence of deleting calcineurin is negligible for this trait (Figure 9). Activation of signaling molecules does not always predict functional consequences of their deletion under equivalent conditions. For example, Mkc1, the mitogen activated protein kinase (MAPK) in the PKC pathway, is activated by hydrogen peroxide but is not required for survival under this condition [43]. Our results suggest that there may be other redundant pathways operating in parallel with Hsp90 and calcineurin in *S. cerevisiae*. The protein kinase C (PKC) cell wall integrity pathway has a well-established function in mediating tolerance to echinocandins in *S. cerevisiae* [44],[45]. In *C. albicans*, the PKC pathway is activated under diverse stress conditions [43] and works in concert with calcineurin and the high osmolarity glycerol pathway to regulate chitin synthesis, which can enhance tolerance to echinocandins [15],[16]. There may be considerable interaction between PKC signaling, calcineurin, and Hsp90. In *S. cerevisiae*, expression of one of the two partially redundant genes encoding the essential (1,3)-β-D-glucan synthase activity, *FKS2*, is regulated by both PKC signaling and calcineurin [46],[47]. In *S. cerevisiae*, Hsp90 may also interact with PKC signaling by chaperoning PKC [48] and the MAPK Slt2 [49],[50].

Stress response signaling and canonical resistance mechanisms are intimately connected in defining a resistance phenotype. Compromising Hsp90 or calcineurin blocks the stress responses crucial for basal tolerance of strains that were not previously exposed to echinocandins (Figures 1, 2, and 3). There is heterogeneity in the phenotypic consequences of compromising these cellular regulators in strains that acquired resistance by mutation in the drug target Fks1 (Figure 7). For some isolates, resistance is not affected (data not shown), while for others resistance is reduced, though not to the extent of a sensitive strain (Figure 7). This suggests that Hsp90 is not required to enable the phenotypic consequences of the mutant Fks1 protein. Rather, in many of the Fks1 mutants, Hsp90 and calcineurin-dependent stress responses contribute to the overall resistance phenotypes. Notably, the calcineurin inhibitor was more effective than the Hsp90 inhibitor at reducing MF resistance of some clinical isolates (Figure 7 and data not shown); this may be due to additional effects of CsA on targets distinct from calcineurin. The accumulation of mutations that reduce the dependence of resistance on Hsp90 is reminiscent of the evolution of azole resistance from Hsp90-dependence towards Hsp90-independence observed in isolates that evolved azole resistance in a human host [21].

Hsp90 chaperones many cellular regulators in addition to calcineurin. High-throughput genomic and proteomic studies suggest that Hsp90 may interact with up to 10% of the *S. cerevisiae* proteome [24]. Thus, Hsp90 is poised to regulate responses to antifungal drugs via other signal transduction pathways governing cellular stress responses. That Hsp90 regulates cellular responses to antifungal drugs targeting both the cell membrane and the cell wall via calcineurin emphasizes the importance of calcineurin as regulator of cellular stress responses. Cases of discordance between the phenotypic effects of compromising Hsp90 versus compromising calcineurin may reflect the relative importance of other Hsp90 client proteins in a particular trait or may reflect specificity of the agents used to inhibit these regulators [51].

Our results suggest that targeting Hsp90 may provide a powerful therapeutic strategy in the treatment of fungal infectious disease. *In vitro*, compromising Hsp90 function enhances the efficacy of echinocandins against isolates that evolved resistance in a human host and against isolates not previously exposed to echinocandins (Figure 1, 3, and 7). In a murine model of disseminated candidiasis, genetic impairment of *HSP90* expression enhances the efficacy of an echinocandin (Figure 8). These findings add a new dimension to combinatorial therapeutic strategies for the treatment of *C. albicans* infections. Our previous work established that genetic reduction of Hsp90 levels enhances the efficacy of fluconazole in a murine model of disseminated *C. albicans* infection [22] and that further genetic depletion of *C. albicans* Hsp90 results in complete clearance of an infection in the murine model [40]. These studies establish firm proof-of-principle of Hsp90 as a therapeutic target. Current Hsp90 inhibitors that are well-tolerated in humans as anti-cancer agents exhibit toxicity in the mouse model in the context of an acute fungal infection [22]. However, in an invertebrate model of fungal pathogenesis, these pharmacological inhibitors of Hsp90 function enhance the efficacy of the two most widely deployed classes of antifungal drugs, azoles and echinocandins, against the two leading fungal pathogens of humans, *Candida albicans* and *Aspergillus fumigatus* [22]. Thus, compromising Hsp90 has broad therapeutic potential in combinatorial therapeutic regimens against fungal infections.

Further support for targeting Hsp90 in antifungal therapy emerges from a recombinant antibody against the *C. albicans* chaperone. This recombinant antibody had therapeutic benefits in a clinical trial in combination with amphotericin B, which targets ergosterol [52]. This antibody also demonstrated synergy with the echinocandin caspofungin in a murine model [53]. The mechanism by which this antibody works, however, is unclear as the antibody is unlikely to be able to cross the fungal cell wall and access the cytosol of intact fungal cells, where Hsp90 regulates calcineurin-dependent signaling governing drug resistance. The antibody may work by influencing host immune responses to the pathogen. Consistent with this thinking, heat-shock proteins are immunodominant antigens for the recognition of many pathogens and play a central role in mediating both innate and adaptive immune responses [54],[55].

Hsp90 has taken center stage as a therapeutic target for diverse diseases including cancer and neurodegeneration. Our findings suggest that Hsp90 may provide a much-needed target for life-threatening fungal infectious disease. Inhibitors of Hsp90 and calcineurin both have potent anti-malarial activity, thus extending their impact to the protozoan parasite *Plasmodium falciparum* [56]. Compromising host Hsp90 function in the context of an acute fungal infection is not well tolerated [22]. Perhaps in a related manner, the utility of calcineurin inhibitors in antifungal therapy has been complicated by their immunosuppressive effects [57]. Thus, the challenge in successfully exploiting this strategy lies in developing fungal selective inhibitors of Hsp90 or in targeting fungal specific components of the Hsp90 chaperone machine. Our findings may point to broader paradigm of targeting fungal stress response pathways in the treatment of life-threatening fungal infectious disease.

## Materials and Methods

### Strains and Culture Conditions

Archives of *C. albicans* and *S. cerevisiae* strains were maintained in at −80°C in 25% glycerol. Strains were grown in either YPD (1% yeast extract, 2% bactopeptone, 2% glucose), YPM (as YPD except with 2% maltose), or in synthetic defined media (yeast nitrogen base, 2% glucose) and supplemented with the required amino acids. 2% agar was added for solid media. Strains were transformed following standard protocols. Strains used in this study are listed in Table S1. Strain construction is described in Text S1.

### Plasmid Construction

Recombinant DNA procedures were performed according to standard protocols. Plasmids used in this study are listed in Table S2. Plasmid construction is described in the Text S1. Plasmids were sequenced to verify the absence of any nonsense mutations. Primers used in this study are listed in Table S3.

### Minimum Inhibitory Concentration and Checkerboard Assays

Antifungal susceptibility was determined in flat bottom, 96-well microtiter plates (Sarstedt) using a modified broth microdilution protocol, as described [21]. Minimum inhibitory concentration (MIC) tests were set up in a total volume of 0.2 ml/well with 2-fold dilutions of micafungin (MF, generously provided by Julia R. Köhler) or caspofungin (CS, generously provided by Rochelle Bagatell). Echinocandin gradients were typically from 2 µg/ml down to 0 with the following concentration steps in µg/ml: 1, 0.5, 0.25, 0.125, 0.0625, 0.03125, 0.015625, 0.0078125, 0.00390625, 0.00195313. For gradients from 16 µg/ml down to 0, the concentration steps in µg/ml were: 8, 4, 2, 1, 0.5, 0.25, 0.125, 0.0625, 0.03125, 0.015625. Cell densities of overnight cultures were determined and dilutions were prepared such that ∼10^3^ cells were inoculated into each well. Geldanamycin (GdA, A.G. Scientific, Inc.) and radicicol (RAD, A.G. Scientific, Inc.) were used to inhibit Hsp90 at the indicated concentrations, and cyclosporin A (CsA, CalBiochem) and FK506 (A.G. Scientific, Inc.) were used to inhibit calcineurin at the indicated concentrations. Checkerboard assays were set up in a total volume of 0.2 ml/well with 2-fold dilutions of MF across the x-axis of the plate and 2-fold dilutions of either GdA or CsA across the y-axis of the plate. Plates were inoculated as with MIC tests. Dimethyl sulfoxide (DMSO, Sigma Aldrich Co.) was the vehicle for GdA, RAD, CsA, and FK506. Sterile water was the vehicle for MF and CS. Plates were incubated in the dark at 30°C for the time period indicated, at which point plates were sealed and re-suspended by agitation. Absorbance was determined at 600 nm using a spectrophotometer (Molecular Devices) and was corrected for background from the corresponding medium. Each strain was tested in duplicate on at least two occasions. MIC data was quantitatively displayed with color using the program Java TreeView 1.1.3 (http://jtreeview.sourceforge.net).

### Spotting Assays

Strains were grown overnight to saturation in YPD and cell concentrations were standardized based on optical density. Five-fold dilutions (from ∼1×10^6^ cells/ml) were spotted onto indicated media using a spotter (Frogger, V&P Scientific, Inc). Plates were photographed after 2 days in the dark at 30°C. All spottings were done in duplicate on at least two separate occasions.

### β-Galactosidase Assays

*C. albicans* cultures were grown overnight in YPD at 30°C with or without 10 µM CsA, 5 µg/ml FK506, 5 µM GdA, or 5 µM RAD. Cells were diluted to OD_600_ of 0.5 and grown at 25°C for 2 h, at which point they were treated with MF, FL, or CaCl_2_, as indicated. *S. cerevisiae* cultures were grown overnight in synthetic defined medium containing ammonium chloride at 30°C with 1 µg/mL FK506 or 5 µM GdA, as indicated. Cells were diluted to OD_600_ of 0.3 and treated with 0.2 M CaCl_2_, FK506, or GdA, as indicated. Cells were grown for 3 hours at 25°C. Protein was extracted as described [46],[58], and protein concentrations were determined by Bradford analysis. β-galactosidase activity was measured using the substrate ONPG (*O*-nitrophenyl-β-D-galactopyranosidase, Sigma Aldrich Co.), as described [46]. β-galactosidase activity is given in units of nanomoles ONPG converted per minute per milligram of protein (Miller Units). Statistical significance was evaluated using GraphPad Prism 4.0.

### Immunoprecipitation

Yeast cultures were grown overnight in YPD at 30°C. Cells were diluted to OD_600_ of 0.2 in 40 ml and grown to mid-log phase. Cells were washed with sterile H_2_0 and resuspended in 500 µl of lysis buffer containing 20 mM Tris pH 7.5, 100 mM KCl, 5 mM MgCl and 20% glycerol, with one protease inhibitor cocktail (complete, EDTA-free tablet, Roche Diagnostics) per 10 ml, 1 mM PMSF (EMD Chemicals) and 20 mM sodium molybdate (Sigma Aldrich Co.) added fresh before use. Cells were transferred to a 2 mL screw-cap tube and the tube was filled, alternating with glass beads and additional lysis buffer until the beads were just below the meniscus at the top of the tube to reduce foaming during bead beating. Cells were disrupted by bead beating twice for 4 minutes with a 10 minute break on ice between cycles. Lysates were recovered by piercing a hole in the bottom of each tube, placing each tube in a larger 14 ml tube, and centrifuging at 1308×g for three 5-minute cycles, recovering the lysates at each interval. Total collected lysates were cleared by centrifugation at 20817×g for 10 minutes at 4°C and protein concentrations were determined by Bradford analysis.

Anti-FLAG immunoprecipitations were done by diluting protein samples to 1 mg/ml in tris-buffered saline with 20 mM sodium molybdate and incubating with anti-FLAG M2 affinity agarose (Sigma Aldrich Co.) that was washed twice with tris-buffered saline prior to use, as per the manufacturer's specifications, at 4°C overnight. Unbound material was removed by three washes with 1 ml tris-buffered saline and protein was eluted by boiling the sample in one volume of 2× sample buffer.

Anti-IgG immunoprecipitations were done by diluting protein samples to 1 mg/ml in lysis buffer with 0.2% tween and incubating with rabbit IgG agarose (Sigma Aldrich Co.) that was washed three times with lysis buffer prior to use, at 4°C overnight. Unbound material was removed by washing six times with 1 ml lysis buffer with 0.1% tween and protein was eluted by boiling the sample in one volume of 2× sample buffer.

### Immune Blot Analysis

Yeast cultures were grown to mid-log phase, protein was extracted as above, and protein concentrations were determined by Bradford analysis. Protein samples were mixed with one-fifth volume of 6× sample buffer, were boiled for 5 minutes, and then separated on a 10% SDS-PAGE gel. Protein was electrotransferred to PVDF membrane (Bio-Rad Laboratories, Inc.) and blocked with 5% skim milk in phosphate buffered saline with 0.1% tween. Blots were hybridized with antibody against CaHsp90 (1∶10000 dilution, [59]), histone H3 (1∶3000 dilution; Abcam ab1791), FLAG (1∶10000, Sigma Aldrich Co.), Hsc82/Hsp82 (1∶5000, [60]), or TAP (1∶5000, Open Biosystems).

### Murine Model of *C. albicans* Infection

Inoculum was prepared as described for injection of 100 µL of a 2×10^6^ CFU/mL suspension [22]. Inoculum concentrations were verified by cell counts and CFU measurements. Male CD1 mice (Charles River Laboratories) age 8 weeks (weight 30–34 g) were infected via the tail vein. For infection with the wild type, the sample sizes were *n* = 6 mice for the untreated group and *n* = 5 mice for the MF treatment group. For the *tetO-HSP90/hsp90Δ* strain the sample sizes were *n* = 7 mice for the untreated group and *n* = 8 for the MF treatment group. An initial dose finding experiment was performed to determine a concentration of MF that would have negligible effect on fungal burden of mice infected with the wild type; a dose of 2 mg/kg MF (Astellas Pharma, Inc; Deerfield, IL) delivered intraperitoneally at one-hour post infection and then daily resulted in clearance of the fungal burden (data not shown), while a dose of 0.2 mg/kg had no significant effect and was chosen as the dose for this study. Mice were observed three times daily for signs of illness and weighed daily. At day 4 following injection, mice were sacrificed by CO_2_ asphyxiation and the left kidney was removed aseptically, homogenized in PBS and serial dilutions plated for determination of kidney fungal burden, as described [22]. CFU values were expressed as CFU/g of tissue, log-transformed and compared using an ANOVA with post-hoc testing of significance between groups (GraphPad Prism 4.0). Murine work was performed under a protocol approved by the Institutional Animal Use and Care Committee at Duke University Medical Center.

## Supporting Information

Text S1 Supplemental Materials and Methods
(0.06 MB DOC)

Figure S1 Hsp90 plays a crucial role in echinocandin tolerance of *Candida albicans*. (A) Pharmacological inhibition of Hsp90 with geldanamycin (GdA) or radicicol (RAD) reduces micafungin (MF) tolerance of *C. albicans* laboratory strains in an MIC assay. Assays were done in rich medium (YPD) at 30°C for 72 hours. Optical densities were averaged for duplicate measurements and normalized relative to MF-free controls (see colour bar). (B) Pharmacological inhibition of Hsp90 with GdA or inhibition of calcineurin with cyclosporine A (CsA) reduces caspofungin (CS) tolerance of *C. albicans* laboratory strains in an MIC assay. Assays were done in rich medium (YPD) at 30°C for 72 hours. Data was analyzed as in part A. (C) Pharmacological inhibition of Hsp90 with GdA or pharmacological inhibition of calcineurin with CsA reduces CS tolerance of *C. albicans* laboratory strains in an MIC assay. Assays were done in RPMI at 30°C for 72 hours. Data was analyzed as in part A. (D) *C. albicans* laboratory strains are susceptible to CS in an E-test. Resistance of standard *C. albicans* laboratory strains to CS is shown on RPMI solid medium. CS test strips (Etest, AB Biodisk) produced a gradient of drug concentration, highest at the top. Plates were incubated at 30°C for 48 hours.
(0.73 MB TIF)

Figure S2 Tagged alleles of *Candida albicans CNA1* and *HSP90* are functional. (A) The *Candida albicans* HIS-FLAG tagged allele of *CNA1* is functional. Cells were spotted in five-fold dilutions (from 1×10^6^ cells/ml) onto solid rich medium with or without CaCl_2_ to assess calcineurin function. The mutant lacking the regulatory subunit of calcineurin required for its activation, Cnb1, is hypersensitive to calcium stress. The strain with its only allele encoding the catalytic subunit of calcineurin C-terminally HIS-FLAG tagged shows no increase in sensitivity to calcium stress, consistent with functionality of the tagged allele. Plates were photographed after 48 hours in the dark at 30°C. (B) The *Candida albicans* TAP-tagged allele of *HSP90* is functional. Cells were spotted as in part A onto solid rich medium to assess function of Hsp90-TAP. Since Hsp90 is essential, the equivalent growth of the strain with its only *HSP90* allele TAP tagged compared to the untagged counterpart indicates functionality of the tagged allele. Plates were photographed after 48 hours in the dark at 30°C.
(1.68 MB TIF)

Table S1 Strains used in this study.
(0.08 MB DOC)

Table S2 Plasmids used in this study.
(0.03 MB DOC)

Table S3 Primers used in this study.
(0.03 MB DOC)
