# Supplementary material for: Hsp90 Governs Echinocandin Resistance in the Pathogenic Yeast Candida albicans via Calcineurin
Source: PLoS Pathog. 2009 Jul 31;5(7):e1000532. doi: 10.1371/journal.ppat.1000532 (PMC2712069; doi:10.1371/journal.ppat.1000532)
Supplement: Text S1 — Supplemental Materials and Methods (0.06 MB DOC) [file ppat.1000532.s001.doc]

**Supplemental Material**

**Materials and Methods**

**Strain Construction**

**CaLC501:** The plasmid pLC340 was digested with *Kpn*I and *Sac*I to liberate the cassette to C-terminally TAP tag the native *HSP90* allele in CaLC239. For NAT resistant transformants, proper integration was verified by PCR using primers oLC313 and oLC319 as well as oLC316 and oLC319. The *SAP2* promoter was induced to drive expression of FLP recombinase to excise the NAT marker cassette.

**CaLC502:** The plasmid pLC340 was digested with *Kpn*I and *Sac*I to liberate the cassette to C-terminally TAP-tag the only *HSP90* allele in CaLC367. For NAT resistant transformants, proper integration was verified by PCR using primers oLC313 and oLC319 as well as oLC316 and oLC319. The *SAP2* promoter was induced to drive expression of FLP recombinase to excise the NAT marker cassette.

**CaLC590:** The plasmid pLC340 was digested with *Kpn*I and *Sac*I to liberate the cassette to C-terminally TAP-tag the only *HSP90* allele in CaLC367. For NAT resistant transformants, proper integration was verified by PCR using primers oLC313 and oLC319 as well as oLC316 and oLC319. The *SAP2* promoter was induced to drive expression of FLP recombinase to excise the NAT marker cassette. Then plasmid pLC353 was digested with *Kpn*I and *Sac*I to liberate the cassette to C-terminally tag *CNA1* with the 6xHISFLAG tag. For NAT resistant transformants, proper integration was verified by PCR using primers oLC342 and oLC295 as well as oLC292 and oLC343. The *SAP2* promoter was induced to drive expression of FLP recombinase to excise the NAT marker cassette.

**CaLC857:** The plasmid pLC353 was digested with *Kpn*I and *Sac*I to liberate the cassette to C-terminally tag *CNA1* with the 6xHISFLAG tag in CaLC239. For NAT resistant transformants, proper integration was verified by PCR using primers oLC342 and oLC275 as well as oLC343 and oLC274. The *SAP2* promoter was induced to drive expression of FLP recombinase to excise the NAT marker cassette.

**CaLC858:** The plasmid pLC353 was digested with *Kpn*I and *Sac*I to liberate the cassette to C-terminally tag *CNA1* with the 6xHISFLAG tag in CaLC367. For NAT resistant transformants, proper integration was verified by PCR using primers oLC342 and oLC275 as well as oLC343 and oLC274. The *SAP2* promoter was induced to drive expression of FLP recombinase to excise the NAT marker cassette.

**CaLC860:** The plasmid pLC350 was digested with *Kpn*I and *Sac*I to liberate the *CNA1* knock out cassette and was transformed into CaLC239. For NAT resistant transformants, proper integration was verified by PCR using primers oLC275 and oLC590 as well as oLC274 and oLC343. The *SAP2* promoter was induced to drive expression of FLP recombinase to excise the NAT marker cassette. The plasmid pLC353 was digested with *Kpn*I and *Sac*I to liberate the cassette to C-terminally tag the other allele of *CNA1* with the 6xHISFLAG tag**.** For NAT resistant transformants, proper integration was verified by PCR using primers oLC342 and oLC275 as well as oLC343 and oLC274. The *SAP2* promoter was induced to drive expression of FLP recombinase to excise the NAT marker cassette. Presence of the deleted allele was verified by PCR using primers oLC433 and oLC436.

**CaLC861:** The plasmid pLC406 was linearized with *Eco*NI to place the *UTR2p*-*lacZ* construct in CAI-4. For Ura+ transformants, proper integration was verified by PCR using primers oLC661 and oLC621.

**CaLC908:** The plasmid pLC350 was digested with *Kpn*I and *Sac*I to liberate the *CNA1* knock out cassette and was transformed into CaLC239. For NAT resistant transformants, proper integration was verified by PCR using primers oLC275 and oLC590 as well as oLC274 and oLC343. The *SAP2* promoter was induced to drive expression of FLP recombinase to excise the NAT marker cassette.

**CaLC909:** The plasmid pLC350 was digested with *Kpn*I and *Sac*I to liberate the *CNA1* knock-out cassette and was transformed into CaLC908 to create a *cna1* null strain. For NAT resistant transformants, proper integration was verified by PCR using primers oLC275 and oLC590 as well as oLC274 and oLC343. The *SAP2* promoter was induced to drive expression of FLP recombinase to excise the NAT marker cassette. Presence of the deleted allele was verified by PCR using primers oLC590 and oLC343 and absence of an intact *CNA1* allele was verified by PCR using primers oLC588 and oLC591.

**CaLC432:** The plasmid pLC329 was digested with *Kpn*I and *Sac*I to liberate the cassette to replace the native *HSP90* promoter with the *MAL2* promoter and was transformed into CaLC367. For NAT resistant transformants, proper integration was verified by PCR using primers oLC308 and oLC275 as well as oLC309 and oLC274. The *SAP2* promoter was induced to drive expression of FLP recombinase to excise the NAT marker cassette.

**CaLC912:** The plasmid pLC353 was digested with *Kpn*I and *Sac*I to liberate the cassette to C-terminally tag *CNA1* with the 6xHISFLAG tag in CaLC432. For NAT resistant transformants, proper integration was verified by PCR using primers oLC342 and oLC275 as well as oLC343 and oLC274. The *SAP2* promoter was induced to drive expression of FLP recombinase to excise the NAT marker cassette.

**ScLC463:** To construct a *cna1∆/cna2∆* double mutant, the BY4742 alpha strain was mated with the BY4741 *cna2∆* deletion mutant. Diploids were sporulated in liquid medium and tetrads were dissected to obtain alpha haploid meiotic progeny. Presence of the *cna2∆* deletion was verified by PCR with primers oLC101 and oLC326. This *cna2∆* alpha strain was then mated to the BY4741 *cna1∆* deletion mutant and haploid meiotic progeny were picked as above. Presence of the *cna2∆* deletion was verified as above and deletion of *cna1∆* was verified with primers oLC101 and oLC102.

**ScLC642:** The plasmid pLC74 was linearized with *Stu*I to place the *CDRE-lacZ* construct at *URA3* in wild-type *S. cerevisiae* W303. For Trp+ transformants, proper integration of the construct was verified by PCR using the primers oLC870 and oLC524.

**Plasmid Construction**

**pLC329:** The *MAL2* promoter was excised from plasmid pLC90 (pAU22, [1]) by digestion with *Not*I and *Sac*II and was cloned into pLC49 at *Not*I and *Sac*II. ~350 base pairs of homology upstream of the *HSP90* promoter was PCR amplified from SC5314 genomic DNA using primers oLC294 and oLC295 and was cloned into pLC49 containing the *MAL2* promoter at *Kpn*I and *Apa*I. ~350 base pairs of homology downstream of the HSP90 promoter was amplified from SC5314 genomic DNA with primers oLC296 and oLC297 and cloned into pLC49 containing the *MAL2* promoter and the upstream homology at *Sac*II and *Sac*I. The cassette to replace the native *HSP90* promoter with the *MAL2* promoter can be excised with *Kpn*I and *Sac*I.

**pLC340:** ~500 base pairs of homology downstream of *HSP90* was PCR amplified from SC5314 genomic DNA using primers oLC318 and oLC319 and cloned into pLC49 (pJK863, [2]) at *Sac*II and *Sac*I. Proper integration was verified by PCR using primers oLC274 and oLC319. ~500 base pairs of homology to the C-terminal end of *HSP90* prior to the stop codon was amplified from SC5314 genomic DNA using primers oLC313 and oLC315. The TAP tag was amplified from genomic DNA isolated from a *S. cerevisiae* strain containing the TAP tag (*HSC82-TAP*, [3]) using oLC316 and oLC317. A fusion PCR was performed to attach the TAP tag to the C-terminal portion of *HSP90* using oLC313 and oLC317. This fusion product was cloned into pLC49 containing the downstream homology at *Kpn*I and *Apa*I. Proper integration was verified by PCR with oLC313 and oLC275. The cassette to C-terminally TAP tag Hsp90 can be excised with *Kpn*I and *Sac*I.

**pLC350:** Homology upstream of *CNA1* before the start codon was PCR amplified from SC5314 genomic DNA using primers oLC433 and oLC434 and was cloned into pLC49 at *Kpn*I and *Apa*I. Proper integration was verified by PCR using primers oLC275 and oLC433. Homology downstream of *CNA1* after the stop codon was amplified from SC5314 genomic DNA with primers oLC435 and oLC436 and was cloned into pLC49 containing the upstream homology at *Sac*II and *Sac*I. Proper integration was verified by PCR using primers oLC274 and oLC436. The cassette to knock out *CNA1* can be excised with *Kpn*I and *Sac*I.

**pLC353:** Homology at the end of *CNA1* immediately before the stop codon was PCR amplified from SC5314 genomic DNA using primers oLC338 and oLC339, which contains the 6xHISFLAG tag within the primer. This product was cloned into pLC49 at *Kpn*I and *Apa*I and proper integration was verified by PCR using primers oLC338 and oLC275. Homology downstream of the coding region of *CNA1* was amplified from SC5314 genomic DNA using primers oLC340 and oLC341 and cloned into pLC49 containing the upstream region of homology at *Sac*II and *Sac*I. Proper integration was verified by PCR using primers oLC341 and oLC274. The cassette to C-terminally 6xHISFLAG tag the catalytic subunit of calcineurin can be excised with *Kpn*I and *Sac*I.

**pLC406:** pLC90 (pAU22, [1]) was digested with *Kpn*I and *Xho*I to liberate the maltose promoter. ~500 base pairs of the *C. albicans* *UTR2* promoter was amplified from SC5314 genomic DNA with oLC616 and oLC617 and was cloned into the pLC90 backbone at *Kpn*I and *Xho*I. Presence of the insert was tested by PCR with oLC616 and oLC617. This plasmid was then digested with *Bam*HI, which is immediately after *lacZ* and before the *MAL2* terminator. ~500 base pairs of the *UTR2* terminator was PCR amplified from SC5314 genomic DNA with oLC618 and oLC660 and was cloned into pLC90 containing the *UTR2* promoter at *Bam*HI. Directionality of the insert was tested by PCR with oLC661 and oLC660. There is an A to G mutation at -41 and an A to G mutation at -200 but these are not within the CDRE. The *CaUTR2p-lacZ* cassette can be linearized for integration using *Eco*NI.

**E-tests**

Resistance of *C. albicans* strains to CS was determined with Etest strips (AB Biodisk) on RPMI solid medium. ~10^5^ cells were plated prior to application of a test strip. Plates were photographed after 48 hours at 30°C in the dark.

**Supplemental References**

1. Uhl MA, Biery M, Craig N, Johnson AD (2003) Haploinsufficiency-based large-scale forward genetic analysis of filamentous growth in the diploid human fungal pathogen *C. albicans*. EMBO J 22: 2668-2678.

2. Shen J, Guo W, Köhler JR (2005) *CaNAT1*, a heterologous dominant selectable marker for transformation of *Candida albicans* and other pathogenic *Candida* species. Infect Immun 73: 1239-1242.

3. Ghaemmaghami S, Huh WK, Bower K, Howson RW, Belle A, et al. (2003) Global analysis of protein expression in yeast. Nature 425: 737-741.

4. Jones T, Federspiel NA, Chibana H, Dungan J, Kalman S, et al. (2004) The diploid genome sequence of *Candida albicans*. Proc Natl Acad Sci U S A 101: 7329-7334.

5. Blankenship JR, Heitman J (2005) Calcineurin is required for *Candida albicans* to survive calcium stress in serum. Infect Immun 73: 5767-5774.

6. Noble SM, Johnson AD (2005) Strains and strategies for large-scale gene deletion studies of the diploid human fungal pathogen *Candida albicans*. Eukaryot Cell 4: 298-309.

7. Cowen LE, Singh SD, Kohler JR, Collins C, Zaas AK, et al. (2009) Harnessing Hsp90 function as a powerful, broadly effective therapeutic strategy for fungal infectious disease. Proc Natl Acad Sci U S A 106: 2818-2823.

8. Shapiro RS, Uppuluri P, Zaas AK, Collins C, Senn H, et al. (2009) Hsp90 orchestrates temperature-dependent *Candida albicans* morphogenesis via Ras1-PKA signaling. Curr Biol 19: 621-629.

9. Onyewu C, Wormley FL, Jr., Perfect JR, Heitman J (2004) The calcineurin target, Crz1, functions in azole tolerance but is not required for virulence of *Candida albicans*. Infect Immun 72: 7330-7333.

10. Giaever G, Chu AM, Ni L, Connelly C, Riles L, et al. (2002) Functional profiling of the *Saccharomyces cerevisiae* genome. Nature 418: 387-391.

11. Winzeler EA, Shoemaker DD, Astromoff A, Liang H, Anderson K, et al. (1999) Functional characterization of the *S. cerevisiae* genome by gene deletion and parallel analysis. Science 285: 901-906.

12. Cowen LE, Lindquist S (2005) Hsp90 potentiates the rapid evolution of new traits: drug resistance in diverse fungi. Science 309: 2185-2189.

13. Balashov SV, Park S, Perlin DS (2006) Assessing resistance to the echinocandin antifungal drug caspofungin in *Candida albicans* by profiling mutations in *FKS1*. Antimicrob Agents Chemother 50: 2058-2063.

14. Stathopoulos AM, Cyert MS (1997) Calcineurin acts through the *CRZ1/TCN1*-encoded transcription factor to regulate gene expression in yeast. Genes Dev 11: 3432-3444.
