# Supplementary figures and images for: Hsp90 Governs Echinocandin Resistance in the Pathogenic Yeast Candida albicans via Calcineurin
Source: PLoS Pathog. 2009 Jul 31;5(7):e1000532. doi: 10.1371/journal.ppat.1000532 (PMC2712069; doi:10.1371/journal.ppat.1000532)

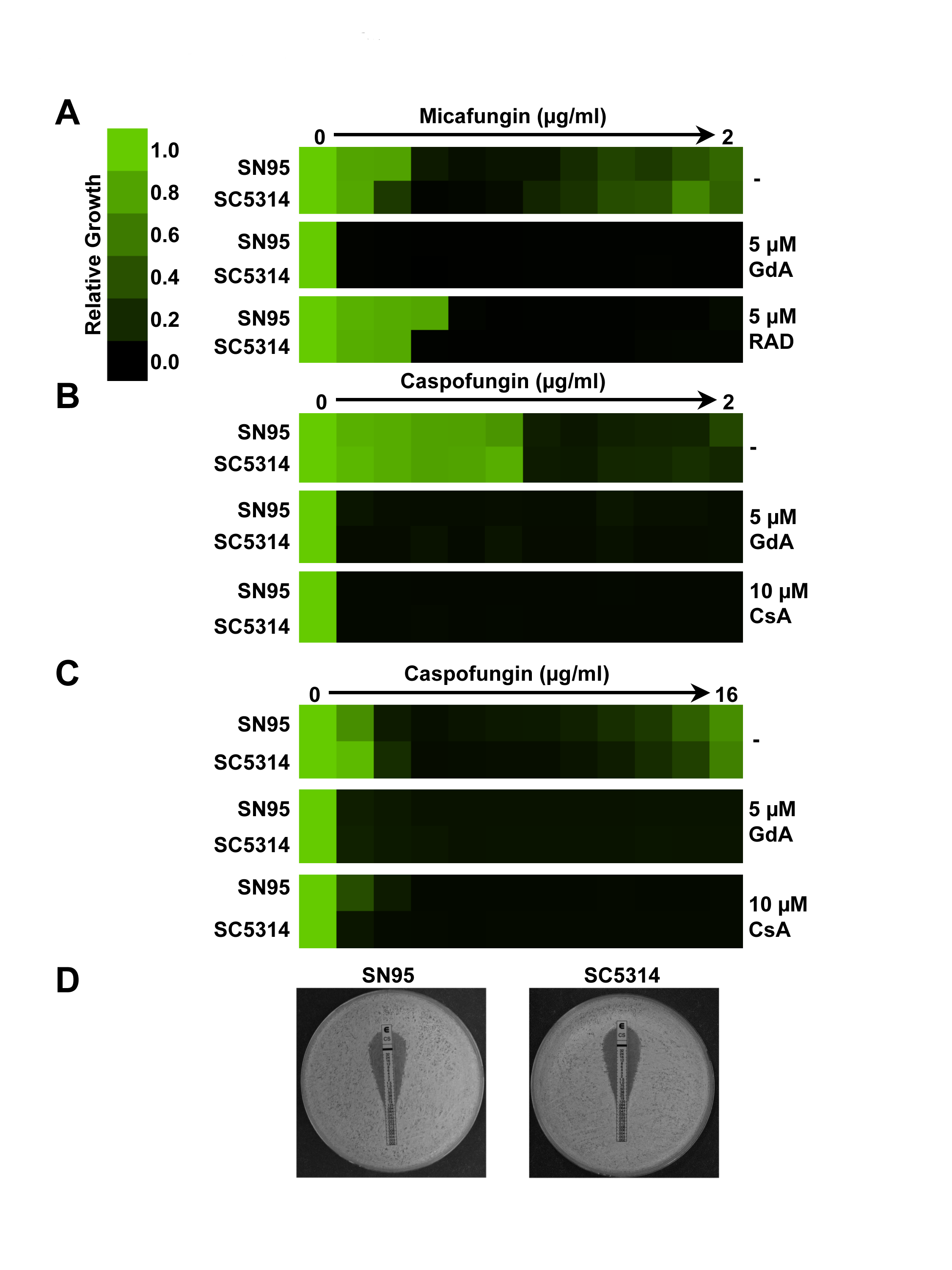

Supplement: Figure S1 — Hsp90 plays a crucial role in echinocandin tolerance of Candida albicans. (A) Pharmacological inhibition of Hsp90 with geldanamycin (GdA) or radicicol (RAD) reduces micafungin (MF) tolerance of C. albicans laboratory strains in an MIC assay. Assays were done in rich medium (YPD) at 30°C for 72 hours. Optical densities were averaged for duplicate measurements and normalized relative to MF-free controls (see colour bar). (B) Pharmacological inhibition of Hsp90 with GdA or inhibition of calcineurin with cyclosporine A (CsA) reduces caspofungin (CS) tolerance of C. albicans laboratory strains in an MIC assay. Assays were done in rich medium (YPD) at 30°C for 72 hours. Data was analyzed as in part A. (C) Pharmacological inhibition of Hsp90 with GdA or pharmacological inhibition of calcineurin with CsA reduces CS tolerance of C. albicans laboratory strains in an MIC assay. Assays were done in RPMI at 30°C for 72 hours. Data was analyzed as in part A. (D) C. albicans laboratory strains are susceptible to CS in an E-test. Resistance of standard C. albicans laboratory strains to CS is shown on RPMI solid medium. CS test strips (Etest, AB Biodisk) produced a gradient of drug concentration, highest at the top. Plates were incubated at 30°C for 48 hours. (0.73 MB TIF) [file ppat.1000532.s002.tif]

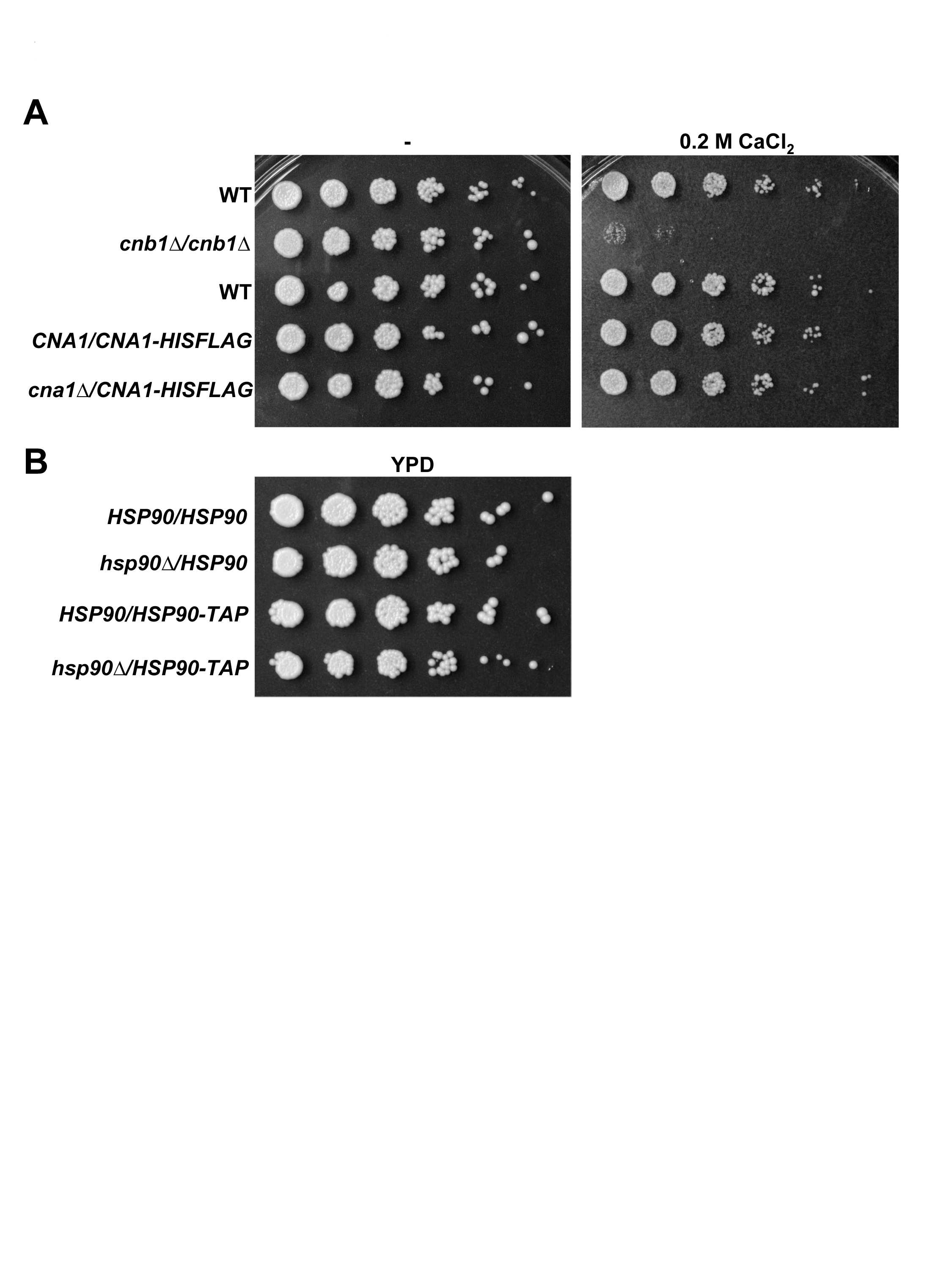

Supplement: Figure S2 — Tagged alleles of Candida albicans CNA1 and HSP90 are functional. (A) The Candida albicans HIS-FLAG tagged allele of CNA1 is functional. Cells were spotted in five-fold dilutions (from 1×10^6^ cells/ml) onto solid rich medium with or without CaCl2 to assess calcineurin function. The mutant lacking the regulatory subunit of calcineurin required for its activation, Cnb1, is hypersensitive to calcium stress. The strain with its only allele encoding the catalytic subunit of calcineurin C-terminally HIS-FLAG tagged shows no increase in sensitivity to calcium stress, consistent with functionality of the tagged allele. Plates were photographed after 48 hours in the dark at 30°C. (B) The Candida albicans TAP-tagged allele of HSP90 is functional. Cells were spotted as in part A onto solid rich medium to assess function of Hsp90-TAP. Since Hsp90 is essential, the equivalent growth of the strain with its only HSP90 allele TAP tagged compared to the untagged counterpart indicates functionality of the tagged allele. Plates were photographed after 48 hours in the dark at 30°C. (1.68 MB TIF) [file ppat.1000532.s003.tif]
