# Supplementary material for: Hsp90 Governs Echinocandin Resistance in the Pathogenic Yeast Candida albicans via Calcineurin
Source: PLoS Pathog. 2009 Jul 31;5(7):e1000532. doi: 10.1371/journal.ppat.1000532 (PMC2712069; doi:10.1371/journal.ppat.1000532)
Supplement: Table S1 — Strains used in this study. (0.08 MB DOC) [file ppat.1000532.s004.doc]

_______________________________________________________________________

Strain Name Genotype Source

_______________________________________________________________________

CaLC155 (SC5314) Prototrophic [4]

CaLC188 *ura3::imm434/ura3::imm434* [5]

*his1::hisG::HIS1/his1::hisG arg4::hisG/arg4::hisG*

*cnb1::UAU1/cnb1::ARG4*

CaLC189 *ura3::imm434/ura3::imm434* [5]

*his1::hisG::HIS1/his1::hisG arg4::hisG/arg4::hisG cnb1::UAU1/cnb1::ARG4*

CaLC190 *ura3::imm434/ura3::imm434* [5]

*his1::hisG::CNB1-HIS1/his1::hisG arg4::hisG/arg4::hisG*

*cnb1::UAU1/cnb1::ARG4*

CaLC191 *URA3/ura3::imm434 HIS1/his1::hisG* [5]

*ARG4/arg4::hisG*

CaLC239 (SN95) *arg4∆/arg4∆ his1∆/his1∆ URA3/ura3::imm434* [6]

*IRO1/iro1::imm434*

CaLC367 As SN95, *HIS1/his1::tetR-FRT* [7]

*hsp90::CdHIS1/HSP90*

CaLC432 As SN95, *HIS1/his1:: tetR-FRT* [8]

*hsp90::CdHIS1/MAL2p-HSP90*

CaLC436 As SN95, *HIS1/his1::TAR-FRT* [7]

*hsp90::CdHIS1/tetO-HSP90*

CaLC501 As SN95, *HSP90/HSP90-TAP-FRT* This Study

CaLC502 As SN95, *HIS1/his1::tetR-FRT* This Study

*hsp90::CdHIS1/HSP90-TAP-FRT*

CaLC587 *crz1∆/crz1∆* [9]

CaLC589 *crz1∆/crz1∆* + *CRZ1* [9]

CaLC590 As SN95, *HIS1/his1::tet-R-FRT* This study

*CNA1/CNA1-HISFLAG-FRT hsp90::CdHIS1/HSP90-TAP-FRT*

CaLC857 As SN95, *CNA1/CNA1-HISFLAG-FRT* This study

CaLC858 As SN95, *CNA1/CNA1-HISFLAG-FRT* This study

*HIS1/his1::tet-R-FRT hsp90::CdHIS1/HSP90*

CaLC860 As SN95, *cna1∆/CNA1-HISFLAG-FRT* This study

CaLC861 (CAI4) *ura3::imm434/ura3::imm434* [8]

*UTR2-UTR2p-lacZ-URA3*

CaLC908 As SN95, *CNA1/cna1::FRT* This study

CaLC909 As SN95, *cna1::FRT/cna1::FRT* This study

CaLC912 As SN95, *HIS1/his1::tet-R-FRT* This study

*hsp90::CdHIS1/MAL2p-HSP90 CNA1/CNA1-HISFLAG-FRT*

CaLC946 As SN95, *HIS1/his1::tet-R-FRT* [7]

*HSP90/FRT-tetO-HSP90*

ScLC151 (BY4741) *his3Δ leu2Δ met15Δ ura3Δ* [10,11]

ScLC400 (Lo90) *can1-100 his3-11,15 leu2-3,112 trp1-1 ura3-1* [12]

*ade2-1 hsc82::KAN; hsp82::KAN; LEP-HSP82-HIS3*, pRS304 (*TRP1*)

ScLC402 *can1-100 his3-11,15 leu2-3,112 trp1-1 ura3-1*  [12]

*ade2-1*, pRS304 (*TRP1*), pRS303 (*HIS3*)

ScLC408 *can1-100 his3-11,15 leu2-3,112 trp1-1 ura3-1*  [12]

*ade2-1 cnb1::KAN*, pRS304 (*TRP1*), pRS303 (*HIS3*)

ScLC463 *his3Δ leu2Δ met15Δ ura3Δ cna1::KAN*  This study

*cna2::KAN*

ScLC642 *can1-100 his3-11,15 leu2-3,112 trp1-1 ura3-1I* This study

*ade2-1 CDRE-lacZ-URA3-TRP*

ScLC863 *his3Δ leu2Δ met15Δ ura3Δ CNA1-TAP-HIS3MX6* [3]

C42 (CaLC986) As SC5314, *FKS1* F641S [13]

DPL15 (CaLC990) Clinical isolate, *FKS1* F641S David Perlin

**______________________________________________________________________________**
