# Supplementary material for: Hsp90 Governs Echinocandin Resistance in the Pathogenic Yeast Candida albicans via Calcineurin
Source: PLoS Pathog. 2009 Jul 31;5(7):e1000532. doi: 10.1371/journal.ppat.1000532 (PMC2712069; doi:10.1371/journal.ppat.1000532)
Supplement: Table S2 — Plasmids used in this study. (0.03 MB DOC) [file ppat.1000532.s005.doc]

­­­­­­­­­­______________________________________________________________________________

Plasmid Description (Backbone) Source

______________________________________________________________________________

pLC49 *FLP-CaNAT*, ampR [2]

pLC74 *CYC1p-CDRE-lacZ*  [14]

pLC329 *MAL2p-CaHSP90*, ampR, NAT (pLC49) [8]

pLC340 *CaHSP90-TAP*, ampR, NAT (pLC49) This study

pLC350 *CaCNA1-*KO, ampR, NAT (pLC49) This study

pLC353 *CaCNA1-HISFLAG*, ampR, NAT (pLC49) This study

pLC406 *CaUTR2p-LacZ*, ampR, *URA3* (pLC90 = pAU22 [1]) [8]

______________________________________________________________________________
