# Supplementary material for: Hsp90 Governs Echinocandin Resistance in the Pathogenic Yeast Candida albicans via Calcineurin
Source: PLoS Pathog. 2009 Jul 31;5(7):e1000532. doi: 10.1371/journal.ppat.1000532 (PMC2712069; doi:10.1371/journal.ppat.1000532)
Supplement: Table S3 — Primers used in this study. (0.03 MB DOC) [file ppat.1000532.s006.doc]

­­­­­­­­­­­­­­Primer Description Sequence

oLC101 KanB CTGCAGCGAGGAGCCGTAAT

oLC102 CNA1-A CTCATTGTTCCGTAAGGATTATCAC

oLC274 pJK863down-F CTGTCAAGGAGGGTATTCTGG

oLC275 pJK863up-R AAAGTCAAAGTTCCAAGGGG

oLC292 pJK863down-F2 GCAAGCTTGATGGAAGTTCC

oLC294 CaHsp90-597-F-KpnI CGGGGTACCGATTTCAGGTTGAAGAATTTGC

oLC295 CaHsp90-250-R-ApaI TTGCGGGCCCCTGTTATAGGTAGTAATATGG

oLC296 CaHsp90-1-F-SacII TCCCCGCGGTATGGCTGACGCAAAAGTTG

oLC297 CaHsp90+348-R-SacI CCCGAGCTCTCAGCACCAGCACTTAAAGC

oLC308 CaHSP90-698-F GATTATTTGCTCACGGAACC

oLC309 CaHsp90+415-R CTTGGACGTGATCAGCAACC

oLC313 CaHsp90 + 1771F – KpnI GGGGTACCAAGGCTCAAGCTTTGAGGACA-

CC

oLC315 TAP + 20R & AACCCGGGGATCCGTCGACCATCAACTTCT-

CaHsp90 + 2121R TCCATAGC

oLC316 CaHsp90 + 2104F & TAP F GCTATGGAAGAAGTTGATG-

GTCGACGGATCCCCGGG

oLC317 TAP + 558R - ApaI TTGCGGGCCCTCACTGATGATTCGCGTCT-

ACTTTCG

oLC318 CaHsp90 + 2125F-SacII TCCCCGCGGACACCAGAAGGGCTACAGTT

oLC319 CaHsp90 + 2467R – SacI CGAGCTCTCTCTATGTTATGTTACTGG

oLC326 CNA2-A (YML057W) GCCCGAGACAAATGAGAAAATGTC

oLC338 CaCNA1 + 1431F – KpnI GGGGTACCCGAAGAAGAGAAGGCTAATGA-

TGG

oLC339 6x HIS-FLAG & TTGCGGGCCCTTATTTATCATCATCATCTTT-

CaCNA1 + 1827R – ApaI ATAATCACCACCGTGGTGGTGGTGGTGGTG-

GACTTTGAGATAATCTTCTTA

oLC340 CaCNA1 + 1831F - SacII TCCCCGCGGACTTTCTTTTGCCCCTGTTT

oLC341 CaCNA1 + 2180R - SacI CGAGCTCGTAAACGTGGTAATCAAATGG

oLC342 CaCNA1 + 1294F TTTAGTGAAGCTGAAATAGG

oLC343 CaCNA1 + 2355R AAGATCTGAGATCTTCTGCC

oLC433 CaCNA – 433F-KpnI GGGGTACCGACGAAATAGTTAATAAAGC

oLC434 CaCNA + 3R-ApaI TTGCGGGCCCCATGATGATAATGGGGAGCC

oLC435 CaCNA+1828F-SacII TCCCCGCGGTAAACTTTCTTTTGCCCCTG

oLC436 CaCNA+2184R-SacI CGAGCTCGGTTGTAAACGTGGTAATCA

oLC524 CYC1-467F – NotI ATAAGAATGCGGCCGCTTCCGTGTGAGA

CGACATCG

oLC588 6xHISFLAG-CaCNA TCCCCGCGGATGCACCACCACCACCACCA-

+4F-SacII CGGTGGTGATTATAAAGATGATGATGATAA-

ATCAGGAAATACTGTTCAACG

oLC590 CaCNA-729F GGTGCTTATACTAGTTGTGC

oLC591 CaCNA+764-R AATTGAGGTGATAATCCTCC

oLC616 CaUTR2-512F-KpnI CGGGGTACCCTAGGAGTAGTTAGTATC

oLC617 CaUTR2-1R-XhoI GctcgagAACAATAGTAGTAATAGTATCG

oLC618 CaUTR2+1414F-BamHI CGCGGATCCAAGTCAATTTAAACATTAAT-

AG

oLC621 CaUTR2+2050R TCGATCAATTAATGTCCC

oLC660 CaUTR2+1952-R-BamHI CGCGGATCCCTTTAGCCCTAGAAGACTTGG

oLC661 CaLacZ+2934-F gaatctgatgctacatgg

oLC870 Cyc1– 42 –R GTATAGTAATTTATGCTGC

______________________________________________________________________________
